# Differential Regulation of Kernel Set and Potential Kernel Weight by Nitrogen Supply and Carbohydrate Availability in Maize Genotypes Contrasting in Nitrogen Use Efficiency

**DOI:** 10.3389/fpls.2020.00586

**Published:** 2020-05-15

**Authors:** Ivan A. Paponov, Martina Paponov, Paolo Sambo, Christof Engels

**Affiliations:** ^1^Division of Food Production and Society, Norwegian Institute of Bioeconomy Research, Ås, Norway; ^2^Department of Agronomy, Food, Natural Resources, Animals and Environment, University of Padova, Legnaro, Italy; ^3^Albrecht Daniel Thaer-Institute of Agricultural and Horticultural Sciences, Plant Nutrition and Fertilisation, Humboldt-Universitat zu Berlin, Berlin, Germany

**Keywords:** kernel set, potential kernel weight, nitrogen, nitrogen use efficiency, maize, sink strength, non-structural carbohydrate

## Abstract

Sub-optimal nitrogen (N) conditions reduce maize yield due to a decrease in two sink components: kernel set and potential kernel weight. Both components are established during the lag phase, suggesting that they could compete for resources during this critical period. However, whether this competition occurs or whether different genotypic strategies exist to optimize photoassimilate use during the lag phase is not clear and requires further investigation. We have addressed this knowledge gap by conducting a nutrient solution culture experiment that allows abrupt changes in N level and light intensity during the lag phase. We investigated plant growth, dry matter partitioning, non-structural carbohydrate concentration, N concentration, and ^15^N distribution (applied 4 days before silking) in plant organs at the beginning and the end of the lag phase in two maize hybrids that differ in grain yield under N-limited conditions: one is a nitrogen-use-efficient (EFFI) genotype and the other is a control (GREEN) genotype that does not display high N use efficiency. We found that the two genotypes used different mechanisms to regulate kernel set. The GREEN genotype showed a reduction in kernel set associated with reduced dry matter allocation to the ear during the lag phase, indicating that the reduced kernel set under N-limited conditions was related to sink restrictions. This idea was supported by a negative correlation between kernel set and sucrose/total sugar ratios in the kernels, indicating that the capacity for sucrose cleavage might be a key factor defining kernel set in the GREEN genotype. By contrast, the kernel set of the EFFI genotype was not correlated with dry matter allocation to the ear or to a higher capacity for sucrose cleavage; rather, it showed a relationship with the different EFFI ear morphology with bigger kernels at the apex of the ear than in the GREEN genotype. The potential kernel weight was independent of carbohydrate availability but was related to the N flux per kernel in both genotypes. In conclusion, kernel set and potential kernel weight are regulated independently, suggesting the possibility of simultaneously increasing both sink components in maize.

## Introduction

Nitrogen (N) is a key nutrient for plant growth and is therefore one of the main factors that can be manipulated to increase crop yields (Erisman et al., 2008). However, extensive application of N has adverse environmental impacts due to N losses in the form of volatilized ammonia, nitrous oxide, and nitrate, a water pollutant (Cameron et al., 2013). Therefore, strategies promoting more efficient N use are important to decrease these N losses. One strategy that can increase N use efficiency (NUE) is to grow plants under a suboptimal N supply, but this requires plant breeding programs that can generate new crop genotypes that produce satisfactory yields under N restriction (Moll et al., 1982; Sattelmacher et al., 1994). In this context, the NUE was defined as the ability of a genotype to realize superior grain yields at low soil N conditions when compared with other genotypes (Sattelmacher et al., 1994). New genotypes can be identified by marker-assisted selection, which can pinpoint genes or QTLs responsible for agriculturally important physiological traits. The identification and characterization of the physiological traits with the highest impact on NUE are important for the successful implementation of marker-assisted selection for breeding of genotypes with high NUE.

In maize, a sub-optimal N supply can decrease yield due to reductions in several key yield components, including the number of ears per plant (Monneveux et al., 2005), kernel number (KN) per ear (Uhart and Andrade, 1995; Andrade et al., 2002; Paponov et al., 2005b), and/or weight of individual kernels (Hisse et al., 2019). Numerous experiments have shown that KN per plant is the main determinant of yield. The KN is established in the period that extends from 2 weeks before to 2 weeks after silking and is controlled by three factors: plant growth (plant dry mass increment) during this critical period, dry mass (DM) partitioning to the ears, and kernel set efficiency (i.e., the number of kernels set per unit DM flux to the ears) (Borras and Vitantonio-Mazzini, 2018). Restrictions in N supply during this critical phase bracketing silking can decrease both plant growth (Andrade et al., 2002) and DM partitioning to the ears (D’Andrea et al., 2008), but these responses can vary depending on the maize genotype. The questions of how different genotypes respond to N limitation and why some genotypes show higher kernel set under sub-optimal N supply remain unanswered and require further investigation.

The potential KN is determined by the number of mature florets on the ear inflorescence and is established well in advance of silking (Gonzalez et al., 2019). KN can be reduced during the lag phase due to failure of kernels to develop from ovaries, termed kernel abortion (Hanft et al., 1986). Under suboptimal levels of N supply, kernel abortion is considered the most sensitive component regulating kernel set (Monneveux et al., 2005). However, the reduction in KN might be also related to delayed emergence of apical silking under stress conditions (Lemcoff and Loomis, 1986, 1994). By contrast, the floret number is insensitive or only weakly affected by N and carbohydrate limitation (Lemcoff and Loomis, 1986; DeBruin et al., 2018), whereas extreme drought stress reduces the floret set (Gonzalez et al., 2019).

Variations in kernel weight (KW) can also strongly affect the crop yield (Borras and Gambin, 2010). The potential KW is established during the 2 weeks period after silking, called the “lag phase,” and is characterized by the number of cells per endosperm and the number of starch granules per cell at the end of the lag phase (Jones et al., 1996). The final KW is determined during the grain filling stage, when the potential KW is realized. Notably, the “lag phase” period is therefore critical for two sink components: the kernel set and the potential KW. The simultaneous establishment of these two sink components assumes that these components would compete for resources (i.e., assimilates), meaning that a higher investment in one component would be offset by a lower investment in the other (Ordonez et al., 2018).

The potential KW, like the KN, is also closely associated with the amount of assimilates available per kernel during the lag phase (Gambin et al., 2006). However, which type of assimilate (i.e., carbohydrates or amino acids) contributes to a higher potential KW is difficult to determine based on field experiments, because carbohydrate and N metabolism share close relationships. Experiments with *in vitro* culture have shown a direct role of N in enhancing potential KW (Cazetta et al., 1999); however, this effect of N has not been tested in intact maize plants. Moreover, the role of genotypic differences in the regulation of potential KW under sub-optimal N supply remains unexplored.

In the present study, we sought to gain insights into the regulation of kernel set and potential KW during the lag phase using hydroponic culture, as this allows rapid decreases or increases in the N level during the lag phase. We used abrupt changes in N level and plant shading to decouple N and carbohydrate fluxes and to provide insights into the use of different resources by sinks under stress conditions. This hydroponic system can serve as a prototype for the development of a phenotyping platform for robust characterization of NUE and can aid in the accurate dissection of different physiological traits that contribute to NUE.

The aim of the present work was to determine whether common or different mechanisms are responsible for the regulation of the two main components of sink capacity (KN and potential KW) in maize when growth is restricted by N and C limitations during the lag phase. A further aim was to elucidate the strategies by which NUE genotypes adapt to sub-optimal N and C conditions to maintain high sink capacity. Specifically, we addressed the question of the importance of carbohydrates, N, or some other unknown factors in establishing the KN and KW sink capacity components. We decoupled the effects of N and carbohydrate availability with an abrupt change in the N (NO_3_^–^) level in the nutrient solution or by an abrupt change in carbohydrate availability by shading the plants, and we estimated the carbohydrate and N status at the critical lag phase stages when the two sink components of KN and potential KW are established.

## Materials and Methods

### Plant Material and Growing Conditions

**FIGURE 1:**
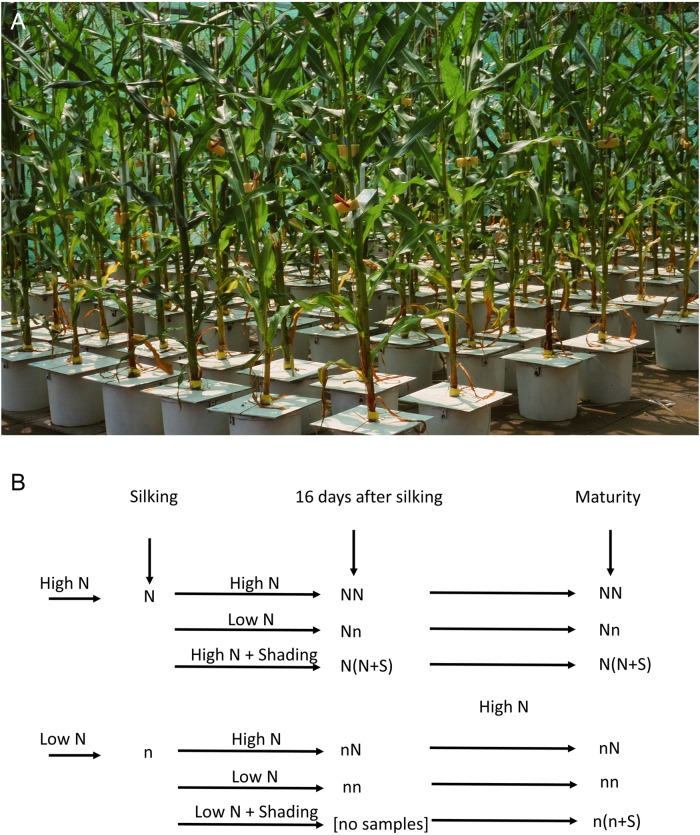
Design of the hydroponics experiment. **(A)** Maize cultivation in 20 L pots in hydroponics with continuous aeration. **(B)** The scheme of the experimental design: high and low N supply before silking, high and low N for 16 days after silking, and shading of the plants under constant N supply; all plants were given excess N and full light during the grain filling stage. Plants were harvested at the beginning of silking, at the end of the lag phase (i.e., 16 days after silking), and at maturity.

The experiment was conducted at the University of Hohenheim (48°43′N, 9°13′E, 407 m altitude) from 26 May (sowing) to 19 September (final harvest) 1997. The average temperature in the summer was 19.8°C, which was 2.9°C warmer than the average values between 1961 and 1990. The sunshine duration reached 822.5 h, which represented 125% of the average values. The plants were cultivated in an area protected from birds by a wire cage but were otherwise exposed to the natural temperature and light conditions (Figure 1A). The experiment compared two maize (*Zea mays* L.) hybrids that had shown similar yields at optimal N supply but different yields at suboptimal N supply in field experiments carried out in 1992 and 1997 (Presterl et al., 2002; Paponov et al., 2005a, b). At suboptimal N supply, the grain yields had been significantly lower for the commercial variety GREEN than for the experimental hybrid EFFI. This latter hybrid had been selected in a breeding program for its high N use efficiency at low levels of N availability (Presterl et al., 2002).

Seeds were germinated for 24 h in 1 mM CaSO_4_ at 25°C under continuous aeration. The kernels were then placed between filter papers fixed with foam and incubated in the darkness. On the 5th day, the emerging seedlings were exposed to light. At day 7, the seedlings were transferred to a dilute nutrient solution containing 10% concentrations of the macronutrients and 100% concentrations of the micronutrients of the standard nutrient solution, along with 0.5 mM of Ca(NO_3_)_2_. At 14 days of age, the seedlings were transferred to 20 L pots containing the standard nutrient solution of 0.1 mM KH_2_PO_5_, 0.5 mM K_2_SO_4_, 0.6 mM MgSO_4_, 150 μM Fe-EDTA, 1 μM H_3_BO_3_, 0.5 μM MnSO_4_, 0.5 μM ZnSO_4_, 0.2 μM CuSO_4_, and 0.01 μM (NH_4_)_6_Mo_7_O_24_. The nutrient solution was changed every 5–7 days. The N supply, in the form of Ca(NO_3_)_2_, varied depending on the treatment. To prevent Ca deficiency, the low-N plants received sufficient CaCl_2_ to provide equivalent Ca to that provided by Ca(NO_3_)_2_ in the high-N treatment. The nutrient solution was maintained between pH 5.5 and 6.5.

The design of the experiment included six treatments (Figure 1B). Plants received the high N supply (N) before silking in three treatments, and the low N supply (n) before silking in three treatments. For both groups of treatments, N supply and light intensity were varied from silking to 16 days after silking. During these 16 days, the plants received either the high N supply (NN, nN) or the low N supply (Nn, nn) to modify the N-nutritional status. The role of plant carbohydrate status on kernel yield was assessed in plants growing in high N status [N(N + S)] or low N status [n(n + S)] by shading the plants to reduce the light intensity by 80%. A lack of a sufficient number of plants prevented sampling for the n(n + S) treatment at the second harvest.

Initially (i.e., after a change of nutrient solution), the nitrate concentration was 0.5 mM in the treatments with high N supply. For this treatment, the nitrate concentration in the nutrient solution was measured daily using the Reflektoquant method (Rqflex, Merck), and nitrate was added regularly to satisfy plant N demand (excluding the complete depletion of nitrate in nutrient solution). With this N supply, plants accumulated about 4948 mg N per plant in the aboveground parts at maturity. This was a significantly higher N uptake than that observed (about 2700 mg per plant) in the aboveground parts in the same genotypes growing in the field under high N supply (Paponov et al., 2005b). These data indicate that N supply in hydroponics was more luxurious than in the field under high N supply. The size of the shoot under these growth conditions was similar to that of the field-grown plants.

In hydroponics, the external concentration is inadequate as a driving variable for N supply because it offers two options for concentration-controlled culture: excess supply and uncontrolled deficiency (Macduff et al., 1993). For this reason, we used a simplified method of N limitation based on relative addition rates, which involved frequent additions of small quantities of nutrient to each pot of nutrient solution (Ingestad and Lund, 1986). This approach was previously applied successfully for comparison of wheat genotypes grown to maturity (Oscarson, 2000). In our study, plants at low N supply until the silking were supplied regularly with 50% of the N amount given to the plants grown under high N supply. During the lag phase, the low N level plants received 200 mg N, whereas N availability was unrestricted for the high N supply plants. Beyond the 16 days after silking and until maturity, all plants received the high N supply to ensure that no limitation of N existed so that the final KW would reflect the potential KW and would not be limited by the growth conditions during the effective grain filling stage.

We used a randomized complete block design with two environmental treatments before silking. During the 16 days of the lag phase, the plants were distributed according to a split-plot design with the main plot being light intensity (shading). We used six environmental treatments during the lag phase. The plant density before the effective grain filling was 4 plants m^–2^. After the end of the lag phase, the plant density was reduced to 3 plants m^–2^ to ensure luxury carbohydrate supply. Rows of border plants were not sampled in this experiment.

### Harvest

Plants were harvested at the beginning of silking, at the end of the lag phase (i.e., 16 days after silking), and at maturity (Figure 1B). One individual plant was used for one replication, with four replications for every time point and every treatment. At the first harvest, the plants were divided into roots, stalk, leaves, ear, and husks. At the second and third harvests, plants were divided into the same organs, but the ear was divided into the cob and kernels. At the end of the lag phase, kernels in a whole row along the rachis of the ear were removed, frozen with liquid nitrogen, and freeze-dried for the analysis of soluble sugars and starch. The other plant organs were dried at 65°C to a constant weight and weighed for dry matter (DM) determination. Leaf weight ratio (LWR), stalk weight ratio (SWR), root weight ratio (RWR), and ear and husk weight ratio (Ear + huskWR) were calculated as DM of leaves, stalk, roots, and ear and husks, respectively, divided by the total DW biomass of the plants. Leaf sheath weights were added to the stalk weights. The plants were harvested according to a randomized complete block design from 10:00 to 12:00, 10:00 to 14:00, and 10:00 to 16:00 for the first, second, and third harvest, respectively. Kernel number was determined for the third harvest by counting all kernels per ear. Individual KW was determined as the ratio between the total kernel weight per ear and the kernel number per ear.

### Soluble Sugars and Starch

A 50 mg sample of ground tissue was mixed with 3.3 mL 70% (v/v) ethanol for sample extraction. Each sample was extracted two more times and sample tubes were centrifuged (3000 × *g*) for 10 min after each extraction. The three supernatants were combined in a test tube and brought to a 10 ml final volume with 70% (v/v) ethanol. Reducing sugars and sucrose were estimated colorimetrically at 415 nm using *p-*hydroxybenzoic acid hydrazide (Blakeney and Mutton, 1980) before and after the digestion of sucrose with invertase for 2 h at 37°C. For starch determination, the ethanol-extracted plant residue was suspended in dimethyl sulfoxide (DMSO) for 10 min (Perez et al., 1971). After centrifugation the supernatant was incubated with amyloglucosidase (2 mL 0.M sodium acetate containing 1.2 U mL^–1^) overnight at 37°C to hydrolyze the starch. The released glucose was then measured colorimetrically at 510 nm (Blakeney and Mutton, 1980) and starch equivalents were calculated.

### Total N and ^15^N Analysis and Calculations

Leaf N content was measured with an automatic N analyzer (Carlo Erba, Milan, Italy, Model 1400). Total plant N uptake was measured as the sum of nitrogen content in different plant parts. The amount of N in different plant parts was calculated by multiplying the dry weight by the N concentration in those parts. Labeled N [^15^N in form of Ca(NO_3_)_2_] was applied at four days before silking. Every plant with low N supply received 53.7 mg (^15^N, 12.1904% excess) and those with high N supply received 55.9 mg (^15^N, 16.8635% excess).

Heavy-isotope concentrations were determined by mass spectrometry (Tracermass, stable isotope analyzer, Europa Scientific) after combustion of the samples in quartz-sealed tubes in the presence of CuO (Roboprep-CN, Europa Scientific). The A% excess was defined as the atom% ^15^N in the plant material minus the atom% in the control sample that did not receive labeled N. The partitioning of recently assimilated N (%P_*N*_) to different organs was determined as previously described (Paponov and Engels, 2005):

%P_*N*_ = (A% excess(^15^N) in organ)/(A% excess (^15^N) in plant) ^∗^(N organ)/(N plant)^∗^100

N flux per ear during the lag phase was measured as the difference between ear N content at the end of the lag phase and ear N content at the beginning of the lag phase.

### Analysis of Ear Morphology in the Field Experiment

The field experiment was conducted in 1998 at the experimental station of the University of Hohenheim Muttergarten in the southern part of Germany (48°43′N, 9°13′E, 407 m altitude). The average temperature in the summer was 20.5°C, which was 3.6°C warmer than the average values between 1961 and 1990. The sunshine duration reached 851.4 h, which represented 129% of the average values. The experiments were randomized block designs with four replications. The soil characteristics, techniques of phosphorus and potassium application, and size of experimental plots were as described previously (Paponov et al., 2005b). Plants were sown on 11 May at a uniform density of 10 plants m^–2^. N fertilizer (calcium ammonium nitrate) was applied at rates of 0 or 150 kg N ha^–1^. Before silk appearance, the ears were covered with paper bags to prevent pollination. The ears were then hand pollinated for two consecutive days, and the paper bags were removed to ensure complete pollination of each ear. Synchronize pollination was used to diminish any differences in pollination time between kernels that might affect on kernel size. Four ears were selected for analysis of the kernel size at different positions along the ear.

### Statistics

The treatments were replicated four times. Data were statistically analyzed by analysis of variance (two-way analysis or three-way analysis, using the ANOVA variables Nv, nitrogen supply before silking, Nf, nitrogen supply during lag phase, Sh, shading during lag phase, G, genotypes). When significant treatment effects were indicated by ANOVA, Fisher’s protected LSD test was used to compare the individual means (Statistica for Windows, version 13).

## Results

### Genotype-Specific Responses to N Supply During the Vegetative Growing Phase: Growth and Dry Matter Partitioning

On average, for both genotypes, a low N supply reduced the plant biomass by 28% when compared with the high N supply (Table 1). Dry mass partitioning among the vegetative plant organs (leaves, stalks, and roots) was not significantly influenced by genotype or rate of N supply, with the exception of a slightly higher leaf weight ratio (LWR) observed at low N than at high N supply. The fact that RWR was not increased at a low N supply was surprising, as the DM partitioning in the roots usually increases under N limitation (Poorter et al., 2012). However, for maize, also other investigations have shown that DM partitioning to the roots is not modulated by the availability of N (Guo and York, 2019). DM partitioning to generative organs, by contrast, was affected differently by N supply in the two genotypes. At the high N supply, DM partitioning to ear 1 and husks (Ear + husk1WR) was greater in the GREEN than in the EFFI genotype, whereas at the low level of N supply, no difference was observed between the two genotypes for DM partitioning to ear 1 and husks. For the GREEN genotype, the DM partitioning to ear 2 and husk (Ear + husk2WR) was reduced to zero at the low level of N supply. By contrast, for the EFFI genotype, the development of ear 2 was not stopped by the low level of N supply.

**TABLE 1:** The influence of N supply up to silking on plant biomass and dry matter distribution at the start of silking for two genotypes with different N-use efficiency.

Nitrogen (N)	Genotype (G)	Biomass, g	LWR	SWR	RWR	Ear + husk1 WR	Ear + husk2 WR
**High**							
	GREEN	129 ± 2^a^	20.1 ± 0.2^a^	45.4 ± 0.9^a^	25.9 ± 1.3^a^	6.99 ± 0.31^b^	1.64 ± 0.45^b^
	EFFI	130 ± 5^a^	20.4 ± 1.0^a^	45.6 ± 2.2^a^	27.9 ± 3.0^a^	3.31 ± 0.32^a^	2.73 ± 0.14^c^
**Low**							
	GREEN	89 ± 1^b^	22.1 ± 0.3^a^	52.0 ± 0.5^a^	22.9 ± 1.0^a^	2.97 ± 0.47^a^	0.00 ± 0.00^a^
	EFFI	98 ± 7^b^	22.0 ± 0.4^a^	45.6 ± 1.7^a^	27.9 ± 2.1^a^	3.35 ± 0.53^a^	1.10 ± 0.08^b^
**ANOVA, source of variation**							
N		***	*	NS	NS	**	***
G		NS	NS	NS	NS	**	***
N × G		NS	NS	NS	NS	**	NS

Dry matter distribution: LWR, leaf weight ratio; SWR, stalk weight ratio; RWR, root weight ratio; Ear + husk1WR, ear 1 and husk weight ratio; Ear + husk2WR, ear 2 and husk weight ratio. Values are means ± SE of four independent biological replicates; different lower case letters behind means indicate significant differences (p ≤ 0.05, Fisher’s protected LSD). *, **, *** Significant at the 0.05, 0.01, and 0.001 probability level, NS, not significant. Factors N level (N), Genotype (G).

### Genotype-Specific Responses to N Supply During the Vegetative Growing Phase: Plant Carbohydrate Status

The concentration of non-structural carbohydrates in the stalks, which was measured as an indicator of plant carbohydrate status, did not differ significantly between the two genotypes (Table 2). Low N decreased the carbohydrate status due to lower sucrose concentrations, but it did not affect the concentrations of reducing sugars and starch. Taken together, the data on N and genotype effects on plant biomass, the ratio of stalk biomass in total plant biomass (SWR), and the non-structural carbohydrate concentrations in the stalks indicated that the amount of reserve carbohydrates in plants at silking was substantially lower in plants given a low N than a high N supply. The genotypes did not differ in their carbohydrate status.

**TABLE 2:** The influence of N supply up to silking on carbohydrate concentration (mg g^–1^) in the stalks at the start of silking for two genotypes with different N-use efficiency.

Nitrogen (N)	Genotype (G)	Red. sugars	Sucrose	Starch
**High**				
	GREEN	66 ± 5^a^	137 ± 9^b^	3.33 ± 0.25^a^
	EFFI	76 ± 8^a^	164 ± 21^b^	2.92 ± 0.33^a^
**Low**				
	GREEN	82 ± 2^a^	40 ± 8^a^	3.86 ± 0.56^a^
	EFFI	72 ± 8^a^	51 ± 11^a^	3.97 ± 0.22^a^
**ANOVA, source of variation**				
N		NS	***	NS
G		NS	NS	NS
N × G		NS	NS	NS

Values are means ± SE of four independent biological replicates; different lower case letters behind means indicate significant differences (p ≤ 0.05, Fisher’s protected LSD). *** Significant at the 0.001 probability level, NS, not significant. Factors N level (N), Genotype (G).

### Genotype-Specific Responses to N Supply During the Vegetative Growing Phase: N Status

Plants under low N supply absorbed about 50% less N than plants under high N supply (Table 3). The N concentrations in the vegetative plant organs were significantly lower in plants supplied with low N than with high N (Table 3). With the high N supply, the N concentrations in leaves and stalks were lower in the EFFI than in GREEN genotype; by contrast, when grown at the low N supply, neither the leaf nor the stalk N concentrations differed between the genotypes. The N concentrations in the generative plant organs were only slightly affected by the N supply rate. The N concentrations in ears 1 and 2 in the EFFI genotype were not significantly reduced by low N compared to high N supply. For the GREEN genotype, the N concentration in ear 1 was even higher at low than at high N supply, which might reflect a dilution effect of N in the ear during development (Ciampitti and Vyn, 2013).

**TABLE 3:** The influence of N supply up to silking on N uptake by plants and N concentrations in plant organs at the start of silking for two maize genotypes with different N-use efficiency.

Nitrogen (N)	Genotype (G)	Plants, mg	Leaf (%)	Stem (%)	Roots (%)	Ear 1 (%)	Ear 2 (%)
**High**							
	GREEN	1906 ± 55^b^	2.71 ± 0.11^c^	0.92 ± 0.03^c^	1.36 ± 0.07^ab^	2.69 ± 0.03^a^	3.93 ± 0.04^b^
	EFFI	1752 ± 66^b^	2.39 ± 0.12^b^	0.76 ± 0.04^b^	1.47 ± 0.11^b^	3.50 ± 0.15^b^	3.09 ± 0.13^a^
**Low**							
	GREEN	893 ± 5^a^	1.63 ± 0.05^a^	0.63 ± 0.02^a^	1.16 ± 0.04^ab^	3.20 ± 0.11^b^	
	EFFI	988 ± 18^a^	1.68 ± 0.03^a^	0.60 ± 0.03^a^	1.04 ± 0.14^a^	3.12 ± 0.13^ab^	3.13 ± 0.18^a^
**ANOVA, source of variation**							
N		***	***	***	*	NS	
G		NS	NS	**	NS	*	
N × G		*	NS	*	NS	**	

Values are means ± SE of four independent biological replicates; different lower case letters behind means indicate significant differences (p ≤ 0.05, Fisher’s protected LSD). *, **, *** Significant at the 0.05, 0.01, and 0.001 probability level, NS, not significant. Factors N level (N), Genotype (G).

We assessed the partitioning of recently acquired N among plant organs by supplying the plants with ^15^N-labeled nitrate 4 days before silking, and we then quantified the percentages of total plant ^15^N in the vegetative and generative plant organs at silking. Partitioning of recently acquired ^15^N among the plant organs was affected by the previous N supply and by the genotype (Table 4). The partitioning of recently acquired N to leaves and stalks, at the expense of N partitioning to roots, was higher for plants supplied with low N than with high N (Table 4). N partitioning to leaves and stalks tended to be higher, and N partitioning to roots lower, for the GREEN than for the EFFI genotype at both levels of N supply. At the high N supply, ^15^N partitioning to ear 1 and husks was greater in GREEN than in EFFI; however, at the low N supply, no differences were found in ^15^N partitioning to ear 1 and husks between the genotypes (Table 4), in agreement with the genotypic differences observed in biomass partitioning (Table 1). For GREEN, ^15^N partitioning to ear 2 and husks was reduced to zero at low N supply. By contrast, for EFFI, ^15^N partitioning to the ear 2 and husks at low and high levels of N supply were similar.

**TABLE 4:** The percentages of total plant ^15^N in the vegetative and generative plant organs at silking after supplying the plants with ^15^N-labeled nitrate four days before silking, as affected by level of N supply for two maize genotypes with different N-use efficiency.

Nitrogen (N)	Genotype (G)	Leaf	Stem	Roots	Ear + husk 1	Ear + husk 2
**High**						
	GREEN	15.0 ± 1.3^a^	28.3 ± 0.4^a^	38.5 ± 0.01^ab^	16.31 ± 0.75^b^	1.84 ± 0.11^b^
	EFFI	13.9 ± 0.9^a^	24.4 ± 2.4^a^	46.8 ± 2.8^b^	9.61 ± 0.68^a^	5.31 ± 0.51^c^
**Low**						
	GREEN	20.8 ± 0.8^b^	39.0 ± 3.0^b^	33.2 ± 2.1^a^	7.07 ± 2.05^a^	0.00 ± 0.00^a^
	EFFI	15.2 ± 0.9^a^	27.9 ± 2.1^a^	40.1 ± 2.5^a^b	11.07 ± 1.11^a^	5.74 ± 0.16^c^
**ANOVA, source of variation**						
N		**	*	*	*	NS
G		**	*	*	NS	***
N × G		NS	NS	NS	**	**

Values are means ± SE of four independent biological replicates; different lower case letters behind means indicate significant differences (p ≤ 0.05, Fisher’s protected LSD). *, **, *** Significant at the 0.05, 0.01, and 0.001 probability level, NS, not significant. Factors N level (N), Genotype (G).

### Genotype-Specific Responses to Modification of N Supply and/or to Shading During the Lag Phase: Growth and DM Partitioning

**FIGURE 2:**
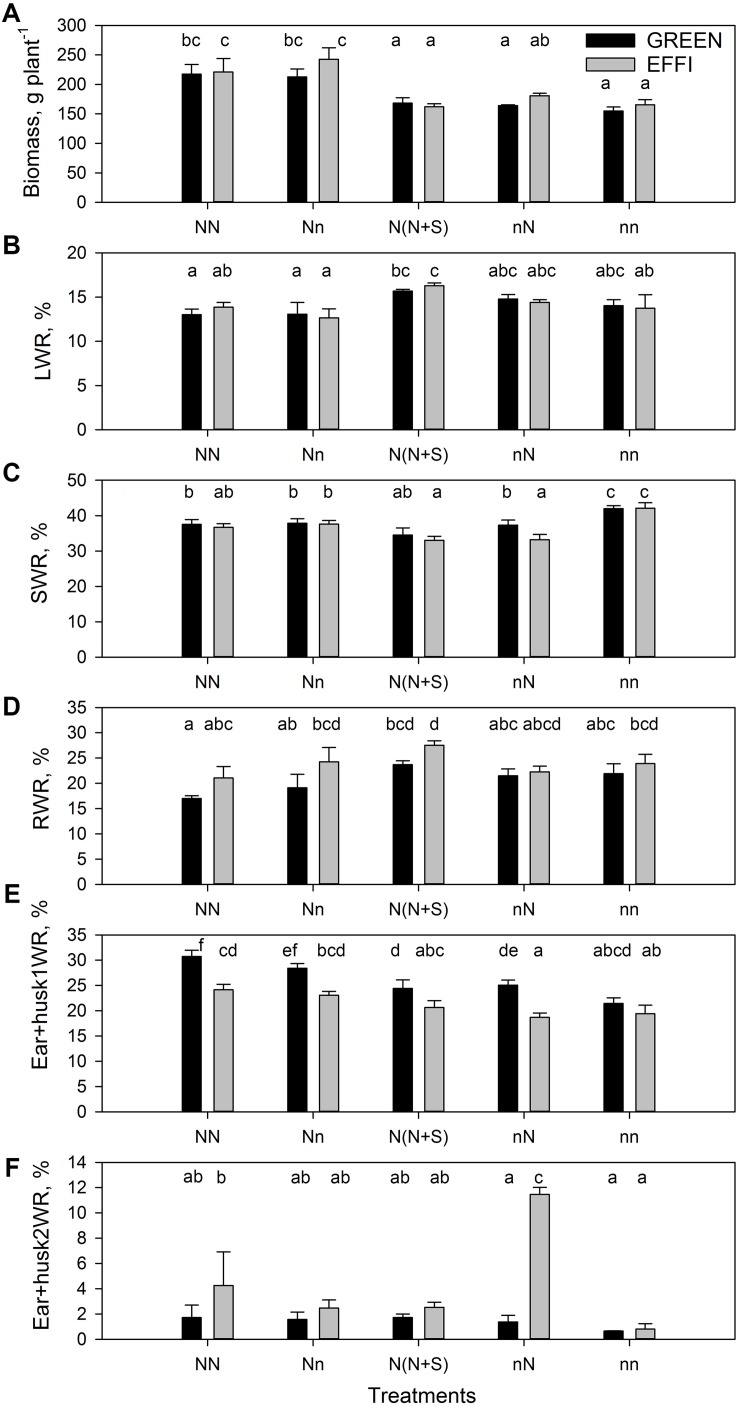
Plant biomass and dry matter distribution in two genotypes with different N-use efficiency at the end of the lag phase. **(A)** Plant biomass, **(B)** leaf weight ratio (LWR), **(C)** stalk weight ratio (SWR), **(D)** root weight ratio (RWR), **(E)** ear 1 and husk weight ratio (Ear + husk1WR), **(F)** ear 2 and husk weight ratio (Ear + husk2WR). Treatments: High N before silking and during the lag phase (NN), high N before silking and low N during the lag phase (Nn), high N before silking and during the lag phase and shading during the lag phase [N(N + S)], low N before silking and high N during the lag phase (nN), low N before silking and during the lag phase (nn). Means ± SE are presented. Different lower case letters indicate significant differences (*p* ≤ 0.05, Fisher’s protected LSD). The data was analyzed by 2-factor-ANOVA. The first factor was treatments (T) [NN, Nn, N(N + S), nN, nn] and the second factor was genotype (G). Excluding shading from the analysis, the experiment was also analyzed by 3-factor ANOVA, using factors Nv, Nf, and G. The results of ANOVAs are presented in Supplementary Table S1.

In the 16 days between silking and the end of the lag phase, the DM per plant increased by about 100 g in plants that were previously (i.e., during the vegetative growing phase) supplied optimally with N, and by 60 g in plants previously supplied with sub-optimal N levels (compare Table 1 and Figure 2A). For both groups of plants, the short-term modification of growing conditions during the lag phase [i.e., the reduction in N supply (Nn) for high N plants and the increase in the N supply for low N plants (nN)] did not significantly change the total plant DM (Figure 2A and Supplementary Table S1).

The level of N supply and the shading during the lag phase had different effects on DM allocation between the vegetative organs: a low N supply during the lag phase increased DM partitioning into the stalks (the main storage organ for carbohydrates) in plants grown at low levels of N before silking but had no effect on DM partitioning in plants grown at high N before silking. These responses indicated a sink limitation in N-deficient plants. By contrast, shading increased DM partitioning into the leaves, in agreement with the common trend of plant adaptation to low light intensity (Poorter et al., 2012) (Figures 2B,C and Supplementary Table S1). This indicates that plants have different mechanisms for adapting to N and carbohydrate limitations. A reduction in the N supply (Nn) or an increase in the N supply for low-N supplied (nN) plants did not change the DM partitioning to the roots, whereas shading [N(N + S)] increased the DM allocation to roots. Analysis of variance showed a significant genotypic effect in the DM partitioning into roots (Supplementary Table S1), as root DM allocation was higher for the EFFI than for the GREEN genotype; however, the genotypic significant difference was not identified under specific treatments (Figure 2D).

In plants grown at high N supply before silking, a low N supply during the lag phase did not significantly change DM partitioning to ear 1 and husks, whereas for the GREEN genotype DM partitioning to ear 1 and husks was reduced by shading. Plants exposed to a continuously low level of N supply before silking and during the lag phase showed decreased DM partitioning to the ear 1 and husks when compared to plants grown at a continuously high N supply. Genotypic differences were also apparent in DM partitioning into the first ear and husks, as the EFFI genotype partitioned less DM to ear 1 and husks in all but one treatment [the same amount was partitioned in the (nn) treatment] (Figure 2E). However, a high N supply during the lag phase caused a stronger increase in DM partitioning into ear 2 and husks in the EFFI than in the GREEN genotype when grown at low N before silking. A similar tendency for higher DM partitioning was also evident in the EFFI versus the GREEN genotype when the plants were grown at high N supply before silking (Figure 2F and Supplementary Table S1).

### Genotype-Specific Response to Modification of N Supply and Shading During the Lag Phase: Carbohydrate Status of Plants and Kernels

**FIGURE 3:**
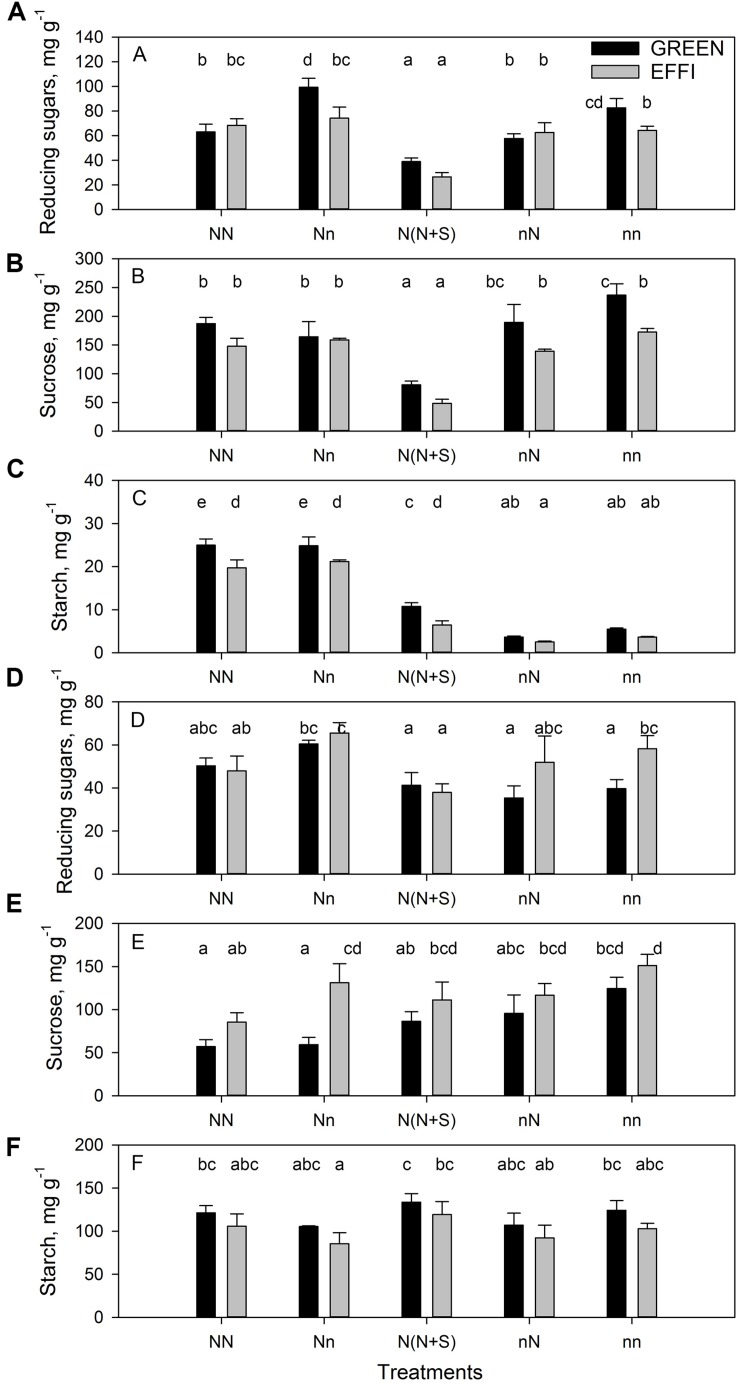
The concentration of carbohydrates in stalk and kernels of two genotypes with different N-use efficiency at the end of the lag phase. Reducing sugars, sucrose, and starch in the stalk **(A–C)** and kernels **(D–F)**. Treatments: High N before silking and during the lag phase (NN), high N before silking and low N during the lag phase (Nn), high N before silking and during the lag phase and shading during the lag phase [N(N + S)], low N before silking and high N during the lag phase (nN), low N before silking and during the lag phase (nn). Means ± SE are presented. Different lower case letters indicate significant differences (*p* ≤ 0.05, Fisher’s protected LSD). The experiment was analyzed by 2-factor-ANOVA. The first factor was treatments (T) [NN, Nn, N(N + S), nN, nn] and the second factor was genotype (G). Excluding shading from the analysis, the experiment was also analyzed by 3-factor ANOVA, using factors Nv, Nf, and G. The results of ANOVAs are presented in Supplementary Table S2.

The whole-plant carbohydrate status at the end of the lag phase, as indicated by the concentrations of non-structural carbohydrates in the stalk, was significantly affected by the rate of N supply before silking (Nv) and by the modifications in the growing conditions during the lag phase (Nf, shading). The response of GREEN plants that were well supplied with N during the vegetative stage to a reduction in the N supply during the lag phase was a significantly increased concentration of reducing sugars, but the EFFI genotype did not show this response (Figure 3A and Supplementary Table S2). The concentrations of sucrose (Figure 3B) and starch (Figure 3C) were not changed in either genotype (compare NN with Nn in Figures 3B,C). Shading drastically diminished the concentrations of reducing sugars, sucrose, and starch in both genotypes [compare NN with N(N + S) in Figures 3A–C].

The response of GREEN plants supplied with sub-optimal N during the vegetative phase to an increase in N supply during the lag phase was a decrease in the concentration of reducing sugars in the stalk, but again the EFFI genotype did not show this response. The concentrations of sucrose and starch in both genotypes were unchanged by the short-term increase in N supply (compare nn with nN in Figures 3A–C). The lower concentration of reducing sugars and starch in the stalks of the EFFI than in the GREEN genotypes under sub-optimal conditions [nn, Nn, N(N + S)] indicates a more efficient carbohydrate utilization at the plant level.

In the kernels, the reduction of N supply during the lag phase (compare NN with Nn) did not change the soluble carbohydrate (sucrose and reducing sugars) concentrations in the GREEN genotype but did increase the sucrose concentration in the EFFI genotype (Figures 3D,E). By contrast, low N supply during lag phase tended to decrease the starch concentration in the kernels in both genotypes, although the difference did not reach statistical significance (Figure 3F). Shading tended to increase the concentration of sucrose but decreased the concentration of reducing sugars in the kernels for both genotypes [compare NN with N(N + S)]. An increased N supply during the lag phase in plants grown at low N supply during the vegetative stage tended to decrease the concentration of sucrose but did not change the concentration of reducing sugars and starch (compare nn with nN). Genotypic comparisons showed that EFFI tended to accumulate higher concentrations of sucrose at both optimal and sub-optimal N conditions (Table 3E and Supplementary Table S2); however, a greater accumulation of reducing sugars in EFFI only occurred in the plants supplied with low levels of N during the vegetative and the lag phases.

### Genotype-Specific Response to Modification of N Supply and Shading During the Lag Phase: N Concentration and Allocation

**FIGURE 4:**
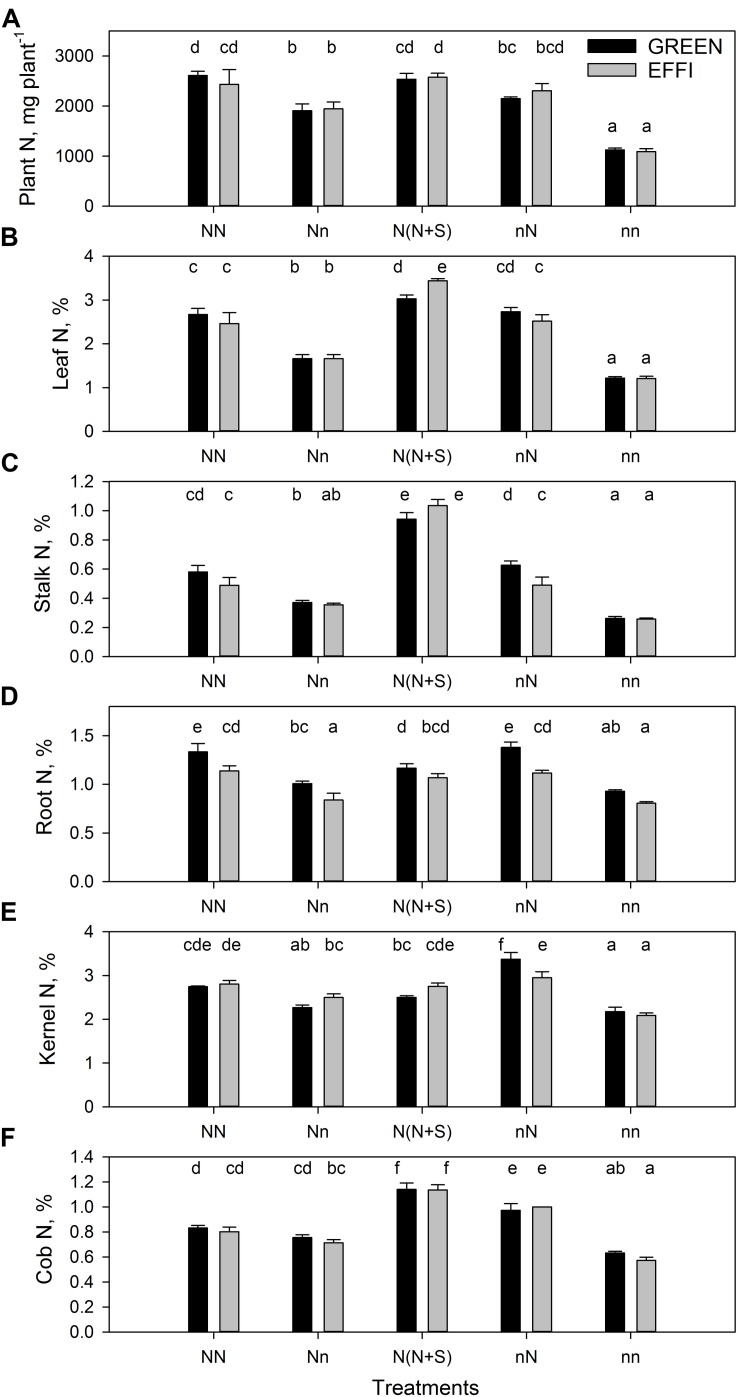
Plant N uptake and N concentration in two genotypes with different N-use efficiency at the end of the lag phase. **(A)** Plant N uptake, N concentration in **(B)** leaves, **(C)** stalk, **(D)** roots, **(E)** kernels, and **(F)** cob. Treatments: High N before silking and during the lag phase (NN), high N before silking and low N during the lag phase (Nn), high N before silking and during the lag phase and shading during the lag phase [N(N + S)], low N before silking and high N during the lag phase (nN), low N before silking and during the lag phase (nn). Means ± SE are presented. Different lower case letters indicate significant differences (*p* ≤ 0.05, Fisher’s protected LSD). The experiment was analyzed by 2-factor-ANOVA. The first factor was treatments (T) [NN, Nn, N(N + S), nN, nn] and the second factor was genotype (G). Excluding shading from the analysis, the experiment was also analyzed by 3-factor ANOVA, using factors Nv, Nf, and G. The results of ANOVAs are presented in Supplementary Table S3.

The change in N supply during the lag phase quickly modulated the N content in plants (Figure 4A and Supplementary Table S3). Low N supply during the lag phase decreased the N concentration in all vegetative organs for plants grown at high N supply before silking (compare NN and Nn); however, low N supply had a weaker effect on N concentration in the generative organs (kernels and cob) (Figures 4B–F and Supplementary Table S3). The N concentration was still higher in these plants than in plants grown at continuously low levels of N (nn). Interestingly, the supply of high N to plants cultivated at a low N level before silking (nN) increased the N concentration to the same level as that seen in plants continuously cultivated at high N. This quick increase in N concentration in these plants indicates a large capacity of the plants to absorb limiting nutrients, as regulated by the demand-driven regulator mechanisms of nitrate uptake (Imsande and Touraine, 1994). Genotypic differences in N concentration were found in the roots (Figure 4D) of plants grown at various N supply conditions (NN, Nn, and nN). The root N concentrations were lower in EFFI than in GREEN, indicating a higher internal N utilization efficiency for root growth. Moreover, under optimal conditions during lag phase, the stalk N concentrations tended to be lower in the EFFI than the GREEN genotype.

**FIGURE 5:**
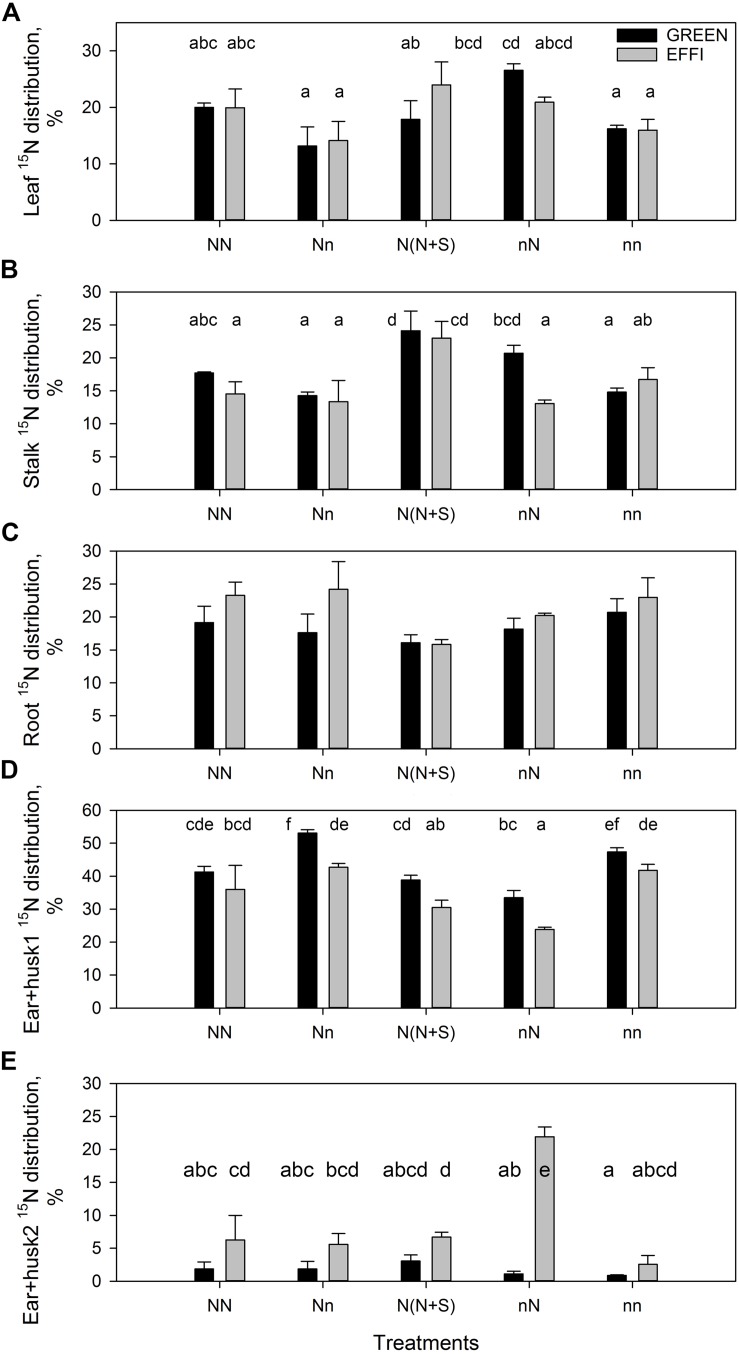
Percentage of ^15^N distribution at the end of the lag phase in two genotypes with different N-use efficiency at the end of the lag phase. ^15^N distribution to **(A)** leaves, **(B)** stalk, **(C)** roots, **(D)** first ear and husks, **(E)** second ear and husks. Treatments: High N before silking and during the lag phase (NN), high N before silking and low N during the lag phase (Nn), high N before silking and during the lag phase and shading during the lag phase [N(N + S)], low N before silking and high N during the lag phase (nN), low N before silking and during the lag phase (nn). Means ± SE are presented. Different lower case letters indicate significant differences (*p* ≤ 0.05, Fisher’s protected LSD). The experiment was analyzed by 2-factor-ANOVA. The first factor was treatments (T) [NN, Nn, N(N + S), nN, nn] and the second factor was genotype (G). Excluding shading from the analysis, the experiment was also analyzed by 3-factor ANOVA, using factors Nv, Nf, and G. The results of ANOVAs are presented in Supplementary Table S4.

Data for the ^15^N distribution showed that low lag phase N supply for plants grown at high N supply during the vegetative stage tended to decrease N partitioning into the leaves. Shading increased N partitioning into the stalk (Figures 5A,B and Supplementary Table S4). The GREEN genotype plants grown at low N supply during the vegetative stage responded to an increased N supply by increasing the N partitioning into the leaves and into the stalk, whereas the EFFI genotype tended to allocate more ^15^N into the roots when grown with a high N supply before silking, regardless of the N supply during the lag phase (Figure 5C). Depletion of N during the lag phase increased ^15^N partitioning into ear 1 and husks, whereas the presence of N during the lag phase increased ^15^N partitioning into ear 2 and husks in the EFFI genotype (Figures 5D,E and Supplementary Table S4). Shading did not significantly affect ^15^N allocation to the ears. The ^15^N distribution to the first ear and husks was lower in the EFFI than in the GREEN genotype under both optimal and sub-optimal conditions and showed significant differences for the Nn, N(N + S), and nN treatments.

### Genotypes Responded Differently in KN to Changes of N and Light Supply During the Lag Phase

**FIGURE 6:**
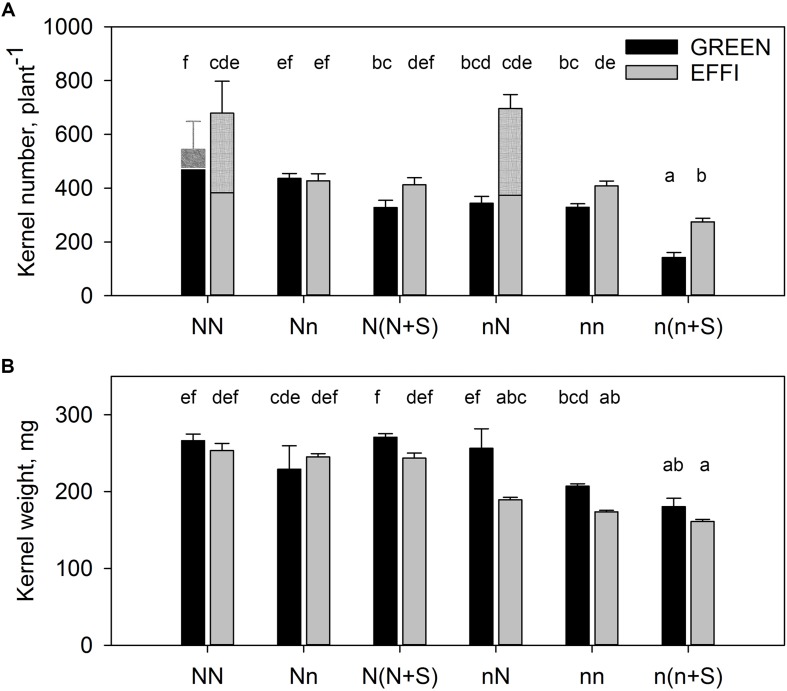
Kernel number **(A)** and kernel weight **(B)** for two genotypes with different N-use efficiency grown under high or low N supply during vegetative growth or during the lag phase and under full light or shading during the lag phase. Treatments: High N before silking and during the lag phase (NN), high N before silking and low N during the lag phase (Nn), high N before silking and during the lag phase and shading during the lag phase [N(N + S)], low N before silking and high N during the lag phase (nN), low N before silking and during the lag phase (nn), and low N before silking and during the lag phase and shading [n(n + S)]. All plants were grown under luxury conditions during the effective grain filling stage to ensure that the final kernel weight corresponded to the potential kernel weight established at the end of the lag phase. Means ± SE are presented. Different lower case letters indicate significant differences (*p* ≤ 0.05, Fisher’s protected LSD). The treatments NN for both genotypes and nN for the EFFI genotype induced formation of a second ear. The kernel number of the second ear is shown in the hatched part of the columns **(A)**, so that the contribution of every ear is presented. The statistical analysis is presented for the first ear. Two separate 3-factor-ANOVAs were carried out. In the first analysis, the factors N level during vegetative stage up to silking (Nv), N level after flowering from silking to 16 days after silking (Nf), and genotype (G) were tested (NN, Nn, nN, nn). In the second analysis, the factors N level (both during vegetative and the lag phase), shading (S), and genotype (G) were tested [NN, N(N + S), nn, n(n + S)]. The results of ANOVAs are presented in Supplementary Table S5.

The KN per plant was higher in EFFI than in GREEN (Figure 6A) because of the formation of the second ear in EFFI under conditions of high N and light during the lag phase. However, a low level of N supply in the lag phase for plants grown under luxury conditions during the vegetative stage did not significantly change KN in the first ear in either genotype (comparing NN vs. Nn) (Figure 6A and Supplementary Table S5). Shading of the plants grown at high N level decreased the KN only in the GREEN and not in the EFFI genotype. Because the floret set is established before silking (Gonzalez et al., 2019), we assume that this kernel number reduction was due to the kernel abortion that occurs during the lag phase. Transfer of plants supplied with low N during the vegetative stage to high N did not increase KN in either genotype; however, shading during the lag phase resulted in a more pronounced decrease in KN in the GREEN than in the EFFI genotype, further indicating that a higher kernel abortion was induced in GREEN than in EFFI under unfavorable conditions during the lag phase. Taken together, the results showed that the EFFI genotype was able to maintain a similar KN under either stress or control conditions, whereas the KN of the GREEN genotype was sensitive to both low N and low light conditions.

### Regulation of KW by N Supply and Shading During the Lag Phase

The mean KW for both genotypes was higher at high than at low N supply during the vegetative stage (Figure 6B and Supplementary Table S5). A reduction in N supply or shading of plants grown under luxury N conditions during the vegetative stage did not decrease the mean KW in either genotype. However, increasing the N supply during the lag phase for plants grown at a low N supply before silking (compare nn vs. nN) increased KW, with a significant effect observed for the GREEN but not for the EFFI genotype. Shading had weak reducing effect on KW in both genotypes.

### Association of KN With N and Carbohydrate Status at the End of the Lag Phase

**FIGURE 7:**
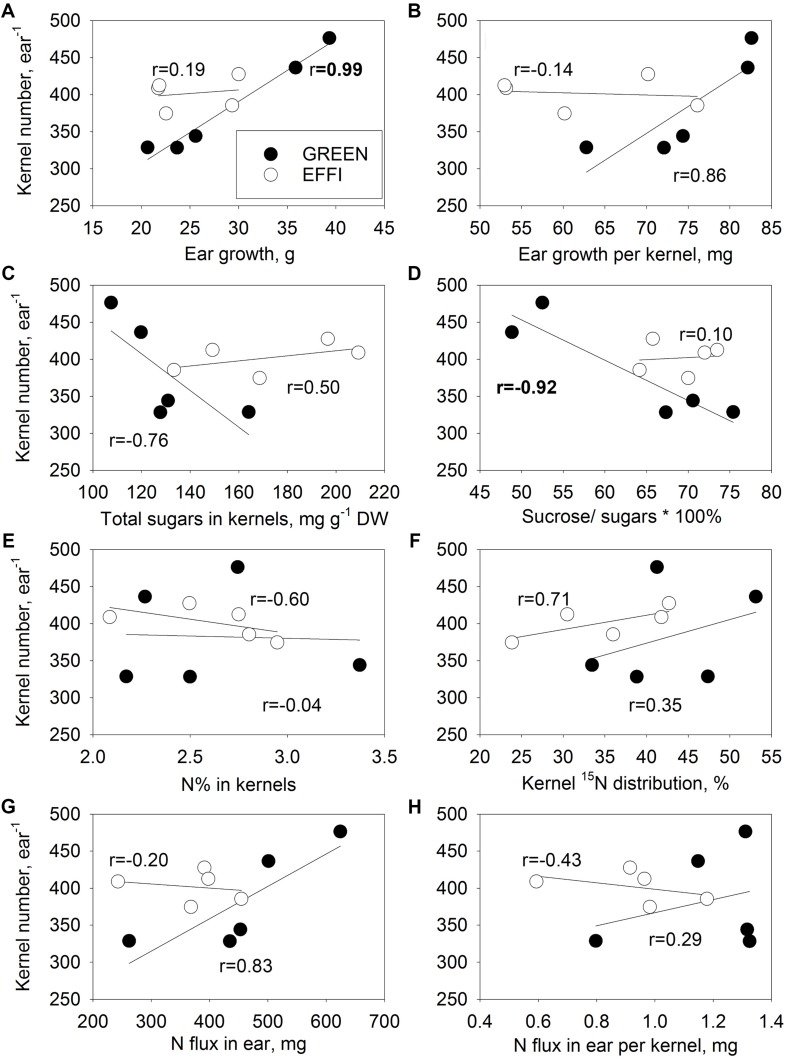
Relationships between kernel number (KN) of the first ear and parameters describing the supply of the first ear with N and carbohydrates during the lag phase for two genotypes with different N-use efficiency. Relationships between KN and ear growth **(A)**, ear growth per kernel **(B)**, total soluble sugar concentrations in kernels **(C)**, ratio of sucrose to total soluble sugar concentrations in kernels **(D)**, kernel N concentrations **(E)**, ^15^N distribution to kernels **(F)**, N flux into ear **(G)**, and N flux into ear per kernel **(H)**. The correlation coefficients (r) in bold are statistically significant at *p* < 0.05.

The reduction in DM partitioning into the ear and the reduced kernel set under stress conditions resulted in a close correlation between these two traits in the GREEN genotype (Figure 7A), whereas for the EFFI genotype, KN was not related to the stress-induced variation in ear growth (Figure 7A). A closer correlation was also observed between ear growth per kernel during the lag phase and KN in the GREEN than in the EFFI genotype (Figure 7B). The GREEN genotype showed a negative correlation between KN and total soluble sugars (Figure 7C) and between KN and the sucrose/sugar ratio (Figure 7D) indicating that, for this genotype, a higher utilization of soluble sugars in the kernels and a higher cleavage of sucrose were related to the higher KN set. Interestingly, the KN for the EFFI genotype was not associated with soluble sugar concentrations in the kernels. The N concentration and fluxes were less associated with KN than was the DM distribution to the ear and KN (Figures 7E–H), indicating that the N supply to the ear was not associated with KN set. The relatively high value of the paired correlation coefficient between KN and N supply to the ear for GREEN (*r* = 0.83) was strongly affected by the high correlation between the DM distribution and N supply to the ear (*r* = 0.89). Indeed, the calculation of the partial correlation coefficient between KN and N supply to the ear show no positive effect of N supply on KN (*r* = −0.69).

### Association of KW With N and Carbohydrate Status at the End of the Lag Phase

**FIGURE 8:**
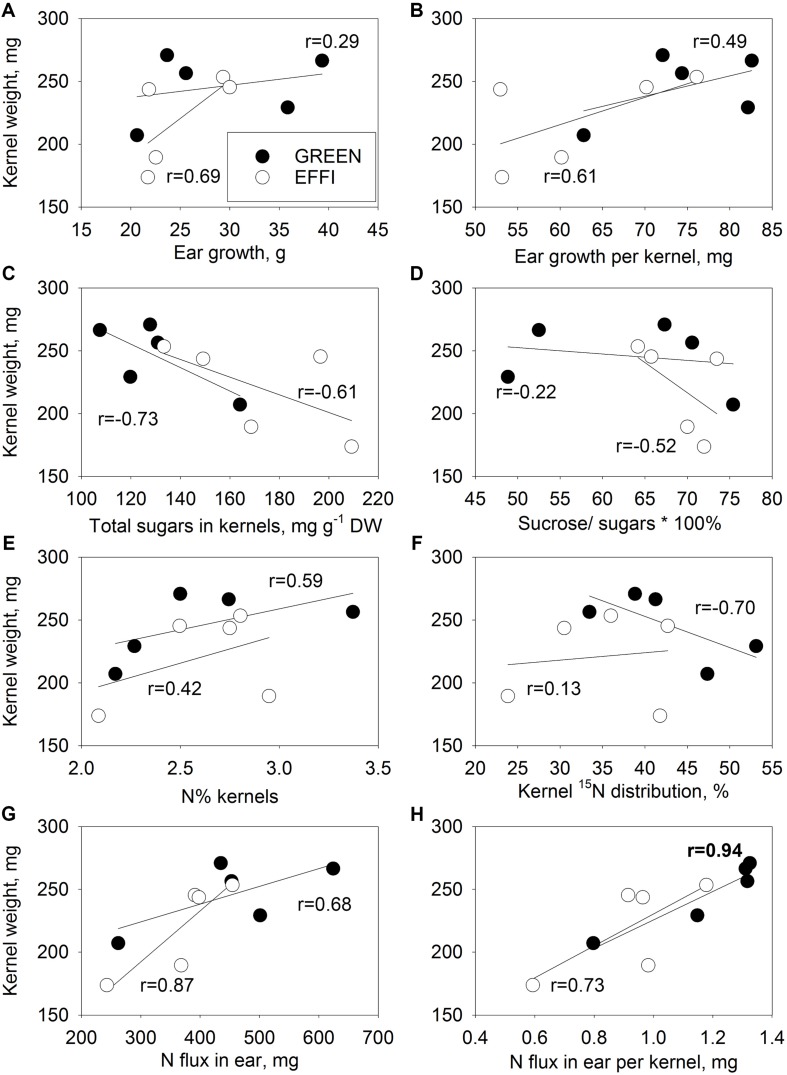
Relationships between kernel weight (KW) of the first ear and parameters describing the supply of the first ear with N and carbohydrate during the lag phase for two genotypes with different N-use efficiency. Relationships between KW and ear growth **(A)**, ear growth per kernel **(B)**, total soluble sugars in kernel **(C)**, ratio of sucrose to total soluble sugars **(D)**, N% kernel **(E)**, ^15^N distribution to kernel **(F)**, N flux into ear **(G)**, and N flux into ear per kernel **(H)**. The correlation coefficients (r) in bold are statistically significant at *p* < 0.05.

No close correlation was detected between KW and ear growth or ear growth per kernel (Figures 8A,B) in either genotype. A weak negative correlation was found between KW and total sugars, indicating that utilization of sugars might be important for both kernel set and KW (Figure 8C). In the GREEN genotype, the correlation between KW and sucrose/total sugar ratio (Figure 8D) was weaker than the correlation between KN and sucrose/total sugar (Figure 7D), indicating that the sucrose cleavage capacity was more important for KN than for potential KW. No strong correlations were found between KW and N% in the kernels and ^15^N distribution (Figures 8E,F); however, closer correlations were found between KW and N flux into the ear and KW and N flux into ear per kernel (Figures 8G,H) for both genotypes, indicating that KW might be associated with N flux to the ear.

### Genotypic Differences in Ear Morphology

**FIGURE 9:**
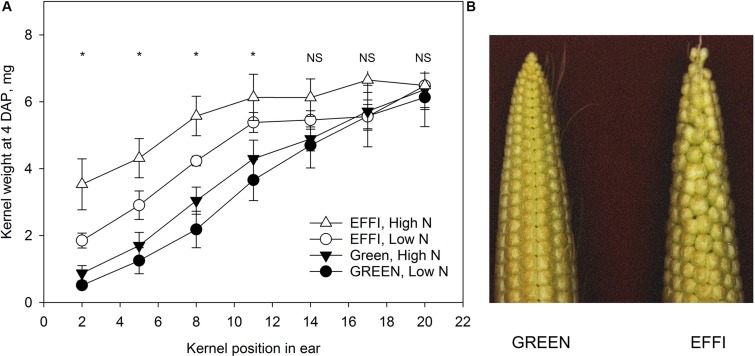
The kernel size four days after pollination for two genotypes with different N-use efficiency grown at high and low level of N supply in a field experiment. **(A)** Kernel dry weight at specific position along the rachis of the ear. Asterisks indicate statistical significant differences among treatments at *p* < 0.05. **(B)** Typical phenotypes of ears of two genotypes at 4 days after pollination.

Analysis of the kernel size at 4 days after pollination (Figure 9) showed that the genotypes differed in the gradient of kernel size: the GREEN genotype showed a strong gradient in kernel size along the ear, with smaller sized kernels at the top and larger sized kernels at the base of ear. By contrast, the EFFI genotype had bigger apical kernels that reduced the gradient of kernel size along the entire ear.

## Discussion

In grain crops, the sink capacity (i.e., the ability of kernels to accumulate assimilates) is determined by the KN and potential KW (Tollenaar, 1977). In maize, both components of sink capacity are fixed during the lag phase of grain growth (Ordonez et al., 2018). Therefore, the processes involved in the determination of KN and potential KW can be assumed to compete for a given pool of resources during this phase, resulting in a restriction of either KN or potential KW. However, if these two components of sink capacity are regulated by different processes and resource pools, then an increase in one component should be possible without a decrease in the other. In the present study, we found indication that these two sink components are regulated independently, implying the possibility of simultaneously increasing both sink components in maize breeding programs.

Independent regulation of KN and potential KW is indicated by the finding that KN and potential KW responded differently to modifications in the supply of either N or carbohydrates during the lag phase in the two genotypes with contrasting N efficiency. The KN per ear (i) was related to the level of available assimilates and their utilization in the kernels during the lag phase in the GREEN genotype, (ii) showed a genotypic difference that was related to morphological traits of the ear determined before silking, and (iii) was regulated by an unknown signal that strongly modulated ear formation and kernel abortion. The potential KW, by contrast, was related to the N flux to the kernels during the lag phase.

### In the Control GREEN Genotype, Kernel Number Was Associated With the Level of Available Assimilates and Their Utilization During the Lag Phase

In the first ear of the GREEN genotype, KN strongly responded to the level of pre-silking N supply and to the light intensity during the lag phase (Figure 6A and Supplementary Table S5). A low pre-silking N supply tend to reduce KN when compared with a high pre-silking N supply. Shading reduced KN for plants with either high N [N(N + S)] or low N supply [n(n + S)]. The tendency toward a reduction in KN under low N supply compared to high N supply before silking indicates a possible inhibitory effect of low N supply on spikelet number; however, limiting conditions during the lag phase have a greater impact on kernel set. Indeed, numerous investigations in maize have shown that KN is closely correlated with crop growth during the lag phase (Andrade et al., 2002; D’Andrea et al., 2009). Crop growth is considered an indicator of a crop’s ability to produce assimilates, i.e., its source strength. In agreement with the well-documented positive relationship between KN and crop growth, all the treatments in the present study that reduced KN also decreased plant growth during the lag phase. The level of N supply during pre-silking and during the lag phase, and the light intensity during the lag phase, also affected N concentrations in various plant organs (Figure 4) and the supply of recently acquired ^15^N to those organs during the lag phase (Figure 5). However, the treatment-induced modification of plant N status and resulting changes of the N delivery to generative organs was not associated with corresponding changes in KN. For example, the abrupt change in N supply from high N during the vegetative growth phase to low N at silking significantly reduced N concentrations in the generative organs, but did not reduce KN. Shading was associated with an increase in plant N concentration, whereas the KN of the GREEN genotype decreased with shading. This suggests that the KN of the GREEN genotype was not directly regulated by the N status of the plants.

Interestingly, experimental conditions that decreased plant growth during the lag phase [nn and N(N + S)] also decreased DM partitioning to the ear (Figure 2). The decrease in DM partitioning to the ear indicates that the sink capacity of the generative plant organs was diminished relative to the sink capacity of other plant organs, resulting in a further reduction of assimilate flux to kernels. This indicates that reduced source strength of the plants was not the only mechanism decreasing kernel set under stress conditions. Under low N and, to a lesser extent, under shading conditions, the concentrations of sucrose in kernels were increased rather than decreased (Figure 3E). Obviously, under these conditions, the sink activity of kernels (i.e., their ability to utilize assimilates for growth and storage) was more depressed than the assimilate supply. This suggestion of lower kernel sink activity under stress conditions is further supported by the increase in the sucrose/total soluble carbohydrate ratio in kernels (Figure 3). This ratio increase indicates that the kernels have a lower sucrose cleavage capacity, which is a crucial factor that determines the high sink activity of kernels. The cleavage of sucrose to hexoses occurs mostly in the pedicel and basal endosperm layer (Shannon, 1972), where apoplastic invertases play a crucial role in establishment of sink strength by maintaining a favorable sucrose concentration gradient between the phloem sieve tubes and the apoplast (Zinselmeier et al., 1999; McLaughlin and Boyer, 2004). A lower ability to metabolize sucrose results in kernel abortion (Bihmidine et al., 2013). The suggestion that plant growth and KN under low N conditions were limited by assimilate utilization (i.e., the sink strength) is also supported by the increased accumulation of hexoses and sucrose in the stalks of GREEN when cultivated at low N (nn) versus high N (NN) conditions (Figures 3A,B).

The suggestion that KN and yield at low N supply are controlled by sink activity rather than source activity is also supported by other investigations. Field experiments on plants grown with different levels of N supply showed that N deficiency increased carbohydrate concentration in the cob (Ning et al., 2018). In that study, N deficiency also increased the ratio of sucrose to total soluble sugars in the apical cob section, where kernel abortion usually occurs. The inhibition of carbohydrate utilization in the kernels was ultimately responsible for feedback inhibition of photosynthesis and of sugar export from leaves under low N supply (Ning et al., 2018). Direct evidence for sink limitation under low N conditions was obtained in a study by Peng et al. (2013), who showed that sucrose infusion into stem internodes in N deficient plants did not increase ear growth.

Some evidence indicates that the low sink strength of ears under N deficiency is related to hormonal signals that are transported to or biosynthesized in the ear. Nitrogen deficiency is associated with lower ear concentrations of IAA and GA during the lag phase and low cytokinin levels at the end of the lag phase, as well as increased concentrations of ABA (Yu et al., 2016). Interestingly, shading during the lag phase induced kernel abortion and induced a similar modulation of hormonal balance, namely lower concentrations of stimulatory hormones, such as IAA, GA and cytokinins, and higher concentration of the inhibitory hormone ABA (Gao et al., 2018).

Other investigations have indicated that ethylene might play a key role in kernel abortion (Cheng and Lur, 1996). A recent investigation on transgenic maize lines with reduced ethylene biosynthesis showed higher KN under different stresses, including shading and N deficiency (Habben et al., 2014). The observations that different types of stresses induce the same changes in hormonal composition in the ear, and that changes in the same signaling pathway increase the yield under different stresses, suggest the operation of a common mechanism for the regulation of kernel set under stress conditions. In our investigation, the observation that KN was higher in EFFI than in GREEN under low N supply and under shading (Figure 6A) further supports the assumption that a common mechanism, involving signaling molecules, might regulate kernel set under different environmental stresses.

In this context, the correlation observed between KN and DM partitioning into the ear (Figure 7) might reflect a feedback loop, whereby a decrease in source strength (assimilate availability) under stress conditions modulates hormone transport to, or hormone biosynthesis in, the ear, resulting in synchronization of sink and source activities. A strategy to restrict kernel set under unfavorable conditions can be seen as being advantageous in maize evolution, as it ensures a sufficient weight and thus amount of reserves in the remaining kernels to support seedling establishment in the following generation. However, this strategy brings no advantages to agriculturally cultivated crops, because a restriction of KN, and thus, sink capacity under unfavorable environmental conditions during the lag phase will strongly decrease final yields.

In this respect, the EFFI genotype is interesting because it was able to maintain a high KN in the first ear even under unfavorable conditions. In this genotype, KN was not related to ear or kernel traits related to assimilate supply to the ear (ear growth during the lag phase, Figure 7A), to assimilate supply to the individual kernels (ear growth per kernel, Figure 7B; total sugar concentrations in kernels, Figure 7C), or to assimilate utilization within the kernels (ratio of sucrose to total sugars in kernels, Figure 7D). The higher KN in the nitrogen use efficient EFFI genotype in comparison to the control GREEN genotype under stress conditions was therefore not related to a higher assimilate supply or to better assimilate utilization by the kernels during the lag phase but was controlled by other plant traits.

### Differences Between Genotypes in Kernel Set Under N Deficiency Are Related to Ear Morphology

The greater ability of EFFI than GREEN to maintain constant KN under low N or shading (Figure 6A) might be related to the differences observed in ear morphology at the beginning of the lag phase (Figure 9). The ears of EFFI contained bigger kernels at the apical part of the ear, resulting in a lower gradient of kernel size along the rachis of the ear. This bigger kernel size at the apical part of the ear might be related with lower dominance of the basal kernels and increase the survival rate of the apical kernels. A smaller kernel size shortly after pollination is associated with a higher probability of kernel abortion in the apical areas (Tollenaar and Daynard, 1978). Moreover, the EFFI genotype, having a shorter ear, might have a more synchronous pollination among silks, which could also contribute to differences in dominance between the basal and apical kernels. Indeed, a reduction in the number of florets per ear should lead to a more uniform development within an ear row and less abortion of apical kernels, as suggested previously (Lafitte and Edmeades, 1995). Recently, genotypic differences were shown in the number of florets and the growth dynamic of kernels at the early stages of cob development in maize, and these differences were related to the capacity of different genotypes to resist abiotic stresses by modulation of kernel abortion (Yan et al., 2018). In that study, the genotype with a higher floret number had a higher rate of kernel loss from the apical position, especially under environmental stress (Yan et al., 2018). Thus, genotypes with a moderate number of florets, but ones of equivalent size, might have the advantages shown the EFFI genotype in our investigation, and this trait could be considered a promising plant characteristic for the selection of genotypes resistant to stress conditions.

The lower kernel size gradient within the cob between apical and basal kernel positions in EFFI in comparison to GREEN was not related to carbohydrate supply to the kernels or to utilization within the kernels (Figures 7A–D). Instead, the larger kernels at the ear apex at the beginning of the lag phase (Figure 9) were more resistant to abortion. Although no advantages were found for carbohydrate utilization in kernels at the end of the lag phase, EFFI had a higher carbohydrate utilization efficiency at the plant level, as indicated by the lower concentration of carbohydrates in the stalk (Figure 3 and Supplementary Table S2). This finding indicates that genotypic differences that lead to more efficient carbohydrate utilization in the stalk can also contribute to the carbohydrate utilization efficiency at the plant level.

The data on N concentrations of different organs in the two genotypes (Figure 4) did not indicate a greater ability of EFFI than GREEN to utilize or redistribute accumulated N. Differences between genotypes in N concentration were found only in the stalk under high N (Table 3), indicating that GREEN might have a higher capacity to accumulate N in the pre-silking stage. This might be advantageous in buffering grain yield under post-silking stress conditions (Nasielski et al., 2019). At the end of the lag phase, EFFI had a lower N concentration in the roots (Figure 4D and Supplementary Table S3), suggesting that the N costs for root growth were lower in EFFI than in GREEN. The lower N concentration in the roots was associated with a higher DM partitioning to the roots, indicating that the more efficient utilization of N for root growth might be an important trait that contributes to the higher NUE of EFFI.

Applying ^15^N 4 days before silking also allowed us to address the question of whether the allocation to various plant organs of the N taken up during the critical period might contribute to more efficient N use. When compared to GREEN, EFFI incorporated more of the ^15^N into the roots and allocated less ^15^N to other vegetative organs, leaves, and the stalk, as well as to the first ear (Table 4). The partitioning of N shortly before silking should be considered in the context of resource competition between the generative organs and roots (Triboi and Triboi-Blondel, 2002). Thus, a higher N investment into the roots might be an important trait of EFFI, as it might contribute to greater root growth, as indicated by the higher DM partitioning into the roots at the end of the lag phase. It might be related to or associated with an alteration in hormone synthesis (Takei et al., 2001), which, in turn, could affect physiological responses in the aboveground plant organs.

### Ear Formation Is Induced by an Unknown Signal

The other indication that unknown signaling molecules might be involved in the regulation of KN per plant is the formation of the second ear in the EFFI genotype following an abrupt increase in N supply at silking (Figure 6A). We found that this abrupt increase in N supply did not significantly modulate plant growth during the lag phase (Figure 2A), indicating that the formation of the second ear was not due to a greater availability of photoassimilates. Interestingly, the formation of the second ear was not typical for the same genotype in the field (Paponov et al., 2005b) and might be related to the low plant density and better N supply used in our hydroponic experiment. The strong increase in sink capacity of the second ear in the EFFI genotype was not associated with genotypic differences in carbohydrate or N concentrations in this genotype when compared with the GREEN genotype. Therefore, we assume that the formation of a second ear was induced by application of nitrate to the roots, which, in turn, stimulated the biosynthesis of phytohormones affecting ear development. The role of hormones in ear initiation has received almost no attention and requires further investigation.

The positive response of the EFFI genotype to high N supply during the lag phase makes this a promising genotype for agronomic management with late N application, which could substantially reduce the losses of applied N occurring through leaching or denitrification, especially in agricultural areas with excessive spring precipitation (Wang et al., 2016). Previous research has generally found that late N application near the critical stage had neither negative nor positive impacts on yield when compared with early season application (Mueller et al., 2017; Mueller and Vyn, 2018b). Interestingly, comparison of modern and 20-year-old genotypes did not show any differences between these two groups in terms of their efficiency at using late applied N fertilizer or with respect to other physiological differences, such as N allocation or DM distribution, among the different organs during the critical period of kernel set establishment (Mueller and Vyn, 2018a). Thus, the differences found here between our two genotypes might be specifically related to the focus of the breeding program, which aimed at breeding genotypes capable of high yield under low N supply conditions.

### Potential KW Is Defined by N Flux per Kernel

The potential KW is fixed at the end of the lag phase, when the number of endosperm cells and the number of starch granules per kernel is established (Jones et al., 1996). However, whether the number of endosperm cells or the number of starch granules is the more important trait that determines the final KW is genotype dependent (Jones et al., 1996). Later, kernel water dynamics have been used as an alternative and easier way than determining endosperm cell number or number of starch granules for estimating potential KW (Borras and Westgate, 2006). The kernel water content at the end of the lag phase provides an indirect estimate of kernel sink capacity (Borras and Gambin, 2010). The evidence that kernel water content at the end of the lag phase is a good predictor of potential KW has been validated by comparing kernel water content with the final KW under optimal conditions during grain filling for a wide range of genotypes and environmental conditions (Borras and Westgate, 2006). Interestingly, for maize, in most cases, the final KW is closely related with the potential size established during the lag phase (Capitano et al., 1983; Reddy and Daynard, 1983; Jones et al., 1996; Borras and Westgate, 2006), so that further optimization of the growth environment (for example, by reduction of plant density) did not increase the final KW. Thus, if no stress conditions occurred during grain filling, the potential kernel weight was usually implemented as the final kernel weight during the grain filling stage.

We cultivated our plants during the effective grain filling stage under luxury conditions (nutrient solution culture with non-limiting supplies of water and nutrients and low numbers of plants m^–2^ ground area to ensure little mutual shading between plants); therefore, we assume that no limitations for effective grain filling existed and that the KW measured at the end of the grain filling stage was very near to the potential KW. Methods involving measurements at the end of the lag phase do not directly measure potential KW; however, they are good predictors of potential KW, as shown previous investigation (Jones et al., 1985; Borras and Westgate, 2006). Nevertheless, the cultivation of plants under luxury conditions during effective grain filling provides a direct way to measure the potential KW, and a direct method can be considered a more robust method for potential KW evaluation.

The GREEN genotype represents an ideal model to study the direct effect of N supply during the lag phase on potential KW because this genotype does not change its KN in the first ear (Figure 6A) in response to changes in N supply. This allows a direct estimation of the effect of N on potential KW in intact plants. Our hydroponic experiment with the abrupt change in N supply showed that N could directly increase the potential KW independent of carbohydrate availability. The evidence for a direct regulation of potential KW by N fluxes is that the abrupt increase in N supply during the lag phase significantly increased the potential KW (Figure 6B and Supplementary Table S5). We assume that this effect is directly related to N and not to carbohydrate availability to the kernels, because a 16-day abrupt change in the N supply did not significantly change the plant biomass (Figure 2A) but strongly increased the N concentration in plant tissues (Figure 4). The close correlation between KW and the amount of N flux per kernel further supports this assumption for both genotypes (Figure 8).

Evidence for direct N effects on the potential KW was also derived from an *in vitro* experiment with kernel explants cultivated in media with varying combinations of N and sucrose (Cazetta et al., 1999). The authors showed that external N, but not sucrose, increased the kernel sink capacity, as indicated by an increased number of cells and starch granules in the endosperm. Numerous field experiments have also shown that both KN and potential KW depend on assimilate availability per kernel during the lag phase (Borras and Westgate, 2006; Gambin et al., 2006, 2008; Severini et al., 2011). However, because of the close link between N and carbohydrate metabolism, the specific effect of N on sink capacity was not resolved. The abrupt change in N availability in our experiment by cultivating plants in hydroponics and shading during the lag phase allowed us to partially dissect the N and carbohydrate fluxes in the plants and to obtain additional indications that N can directly contribute to the potential KW. These results are also supported by the recent observation that a high rate of N increases the number of endosperm cells realized during the lag phase and is associated with the final kernel weights at maturity (Olmedo Pico et al., 2019). This effect was stronger than the increase in carbohydrate availability due to reduced plant density (Olmedo Pico et al., 2019).

Compared to GREEN, EFFI had almost twice the number of kernels per plant after the application of N to the nutrient solution; this difference increases the challenge to elucidate the direct effect of N on potential KW in this genotype. Interestingly, for EFFI, the level of N supplied before silking contributed to the potential KW (Figure 6B). This finding that conditions before silking might affect KW agrees with the consensus that the period 15 days before silking is important for establishing the potential KW (Ordonez et al., 2018). Suboptimal conditions before flowering affect the size of the ovary in wheat, and the size of the ovary affects the final KW (Calderini et al., 1999). However, in maize, the effects of suboptimal conditions before silking require more attention in the future.

## Conclusion

The main finding of this work is that KN and potential KW are regulated by different resources, despite their simultaneous establishment. KN is regulated differently in genotypes that differ in their ability to build grain yield at a low level of N supply. For the standard GREEN genotype, transient shading or a low pre-silking N supply significantly reduced KN, thereby showing a close positive relationship between KN and carbohydrate flux to the ear during the lag phase. The regulation of KN would ensure that the kernels actually established are adequately supplied with organic and inorganic nutrients during grain filling. This growth strategy includes kernel abortion, which leads to fewer kernels but produces kernels that have a better ability to support next-generation seedlings with kernel-derived resources. By contrast, the EFFI genotype, which was selected for its high NUE in a breeding program (Presterl et al., 2002), was characterized by its maintenance of a high KN in the first ear, even under unfavorable N supply or shading conditions, indicating no association between KN and carbohydrate flux to the ear during the lag phase in this genotype. Moreover, under high N supply during the lag phase, the EFFI genotype was able to build the second ear, thereby almost doubling the KN. We assume that this genotype has several advantages for sustainable agriculture as it can build higher sink capacity under both favorable and unfavorable conditions during the lag phase; however, the cultivation of this genotype increases the risk of ending up with small reserves per kernel if growing conditions during grain filling are unfavorable for sufficient resource supply. These different genotypic responses to low N supply and shading during critical kernel formation stages were related to genotypic differences in ear morphology, as the efficient genotype had bigger apical kernels, indicating a reduction in the usual dominance of basal over apical kernels in the control genotype. In contrast to KN, the regulation of potential KW appeared to operate similarly in both contrasting genotypes, as KW was related to the amount of N flux per kernel during the lag phase.

## Data Availability Statement

All datasets generated for this study are included in the article/Supplementary Material.

## Author Contributions

IP and CE conceived and designed the study. IP, MP, and CE wrote the manuscript. IP, MP, and PS performed the experiment. IP and MP performed the analyses.

## Conflict of Interest

The authors declare that the research was conducted in the absence of any commercial or financial relationships that could be construed as a potential conflict of interest.

## References

[B1] Andrade F. H. Echarte L. Rizzalli R. Della Maggiora A. Casanovas M. (2002). Kernel number prediction in maize under nitrogen or water stress. *Crop Sci.* 42 1173–1179. 10.2135/cropsci2002.1173

[B2] Bihmidine S. Hunter C. T. Johns C. E. Koch K. E. Braun D. M. (2013). Regulation of assimilate import into sink organs: update on molecular drivers of sink strength. *Front. Plant Sci.* 4:177. 10.3389/fpls.2013.00177 23761804 PMC3671192

[B3] Blakeney A. B. Mutton L. L. (1980). A simple colorimetric method for the determination of sugars in fruit and vegetables. *J. Sci. Food Agr.* 31 889–897. 10.1002/jsfa.2740310905

[B4] Borras L. Gambin B. L. (2010). Trait dissection of maize kernel weight: Towards integrating hierarchical scales using a plant growth approach. *Field Crop. Res.* 118 1–12. 10.1016/j.fcr.2010.04.010

[B5] Borras L. Vitantonio-Mazzini L. N. (2018). Maize reproductive development and kernel set under limited plant growth environments. *J. Exp. Bot.* 69 3235–3243. 10.1093/jxb/erx452 29304259

[B6] Borras L. Westgate M. E. (2006). Predicting maize kernel sink capacity early in development. *Field Crop. Res.* 95 223–233. 10.1016/j.fcr.2005.03.001

[B7] Calderini D. F. Abeledo L. G. Savin R. Slafer G. A. (1999). Effect of temperature and carpel size during pre-anthesis on potential grain weight in wheat. *J. Agr. Sci.* 132 453–459. 10.1017/s0021859699006504

[B8] Cameron K. C. Di H. J. Moir J. L. (2013). Nitrogen losses from the soil/plant system: a review. *Ann. Appli. Biol.* 162 145–173. 10.1111/aab.12014

[B9] Capitano R. Gentinetta E. Motto M. (1983). Grain weight and its components in maize inbred lines. *Maydica* 28 365–379.

[B10] Cazetta J. O. Seebauer J. R. Below F. E. (1999). Sucrose and nitrogen supplies regulate growth of maize kernels. *Ann. Bot.* 84 747–754. 10.1006/anbo.1999.0976

[B11] Cheng C. Y. Lur H. S. (1996). Ethylene may be involved in abortion of the maize caryopsis. *Physiol. Plant.* 98 245–252. 10.1034/j.1399-3054.1996.980205.x

[B12] Ciampitti I. A. Vyn T. J. (2013). Grain nitrogen source changes over time in maize: a review. *Crop Sci.* 53 366–377. 10.2135/cropsci2012.07.0439

[B13] D’Andrea K. E. Otegui M. E. Cirilo A. G. (2008). Kernel number determination differs among maize hybrids in response to nitrogen. *Field Crop. Res.* 105 228–239. 10.1016/j.fcr.2007.10.007

[B14] D’Andrea K. E. Otegui M. E. Cirilo A. G. Eyherabide G. H. (2009). Ecophysiological traits in maize hybrids and their parental inbred lines: phenotyping of responses to contrasting nitrogen supply levels. *Field Crop. Res.* 114 147–158. 10.1016/j.fcr.2009.07.016

[B15] DeBruin J. L. Hemphill B. Schussler J. R. (2018). Silk development and kernel set in maize as related to nitrogen stress. *Crop Sci.* 58 2581–2592. 10.2135/cropsci2018.03.0160

[B16] Erisman J. W. Sutton M. A. Galloway J. Klimont Z. Winiwarter W. (2008). How a century of ammonia synthesis changed the world. *Nat. Geosci.* 1:636 10.1038/ngeo325

[B17] Gambin B. L. Borras L. Otegui M. E. (2006). Source-sink relations and kernel weight differences in maize temperate hybrids. *Field Crop. Res.* 95 316–326. 10.1016/j.fcr.2005.04.002

[B18] Gambin B. L. Borras L. Otegui M. E. (2008). Kernel weight dependence upon plant growth at different grain-filling stages in maize and sorghum. *Aust. J. Agr. Res.* 59 280–290. 10.1071/ar07275

[B19] Gao J. Shi J. G. Dong S. T. Liu P. Zhao B. Zhang J. W. (2018). Grain development and endogenous hormones in summer maize (Zea mays L.) submitted to different light conditions. *Int. J. Biometeorol.* 62 2131–2138. 10.1007/s00484-018-1613-4 30244320

[B20] Gonzalez V. H. Lee E. A. Lukens L. L. Swanton C. J. (2019). The relationship between floret number and plant dry matter accumulation varies with early season stress in maize (Zea mays L.). *Field Crop. Res.* 238 129–138. 10.1016/j.fcr.2019.05.003

[B21] Guo H. C. York L. M. (2019). Maize with fewer nodal roots allocates mass to more lateral and deep roots that improve nitrogen uptake and shoot growth. *J. Exp. Bot.* 70 5299–5309. 10.1093/jxb/erz258 31145788 PMC6793442

[B22] Habben J. E. Bao X. M. Bate N. J. DeBruin J. L. Dolan D. Hasegawa D. (2014). Transgenic alteration of ethylene biosynthesis increases grain yield in maize under field drought-stress conditions. *Plant Biotechnol. J.* 12 685–693. 10.1111/pbi.12172 24618117

[B23] Hanft J. M. Jones R. J. Stumme A. B. (1986). Dry-matter accumulation and carbohydrate concentration patterns of field-grown and invitro cultured maize kernels from the tip and middle-ear positions. *Crop Sci.* 26 568–572. 10.2135/cropsci1986.0011183X002600030029x

[B24] Hisse I. R. D’Andrea K. E. Otegui M. E. (2019). Source-sink relations and kernel weight in maize inbred lines and hybrids: responses to contrasting nitrogen supply levels. *Field Crop. Res.* 230 151–159. 10.1016/j.fcr.2018.10.011

[B25] Imsande J. Touraine B. (1994). N-demand and the regulation of nitrate uptake. *Plant Phys.* 105 3–7. 10.1104/pp.105.1.3 12232181 PMC159322

[B26] Ingestad T. Lund A. B. (1986). Theory and techniques for steady state mineral nutrition and growth of plants. *Scand. J. Forest Res.* 1 439–453. 10.1080/02827588609382436

[B27] Jones R. J. Roessler J. Ouattar S. (1985). Thermal environment during endosperm cell-division in maize: effects on number of endosperm cells and starch granules. *Crop Sci.* 25 830–834. 10.2135/cropsci1985.0011183X002500050025x

[B28] Jones R. J. Schreiber B. M. N. Roessler J. A. (1996). Kernel sink capacity in maize: genotypic and maternal regulation. *Crop Sci.* 36 301–306. 10.2135/cropsci1996.0011183X003600020015x

[B29] Lafitte H. R. Edmeades G. O. (1995). Stress tolerance in tropical maize is linked to constitutive changes in ear growth characteristics. *Crop Sci.* 35 820–826. 10.2135/cropsci1995.0011183X003500030031x

[B30] Lemcoff J. H. Loomis R. S. (1986). Nitrogen influences on yield determination in maize. *Crop Sci.* 26 1017–1022. 10.2135/cropsci1986.0011183X002600050036x

[B31] Lemcoff J. H. Loomis R. S. (1994). Nitrogen and density influences on silk emergence, endosperm development, and grain-yield in maize (*Zea mays* L.). *Field Crop. Res.* 38 63–72. 10.1016/0378-4290(94)90001-90009

[B32] Macduff J. H. Jarvis S. C. Larsson C. M. Oscarson P. (1993). Plant growth in relation to the supply and uptake of NO3-: a comparison between relative addition rate and external concentration as driving variables. *J. Exp. Bot.* 44 1475–1484. 10.1093/jxb/44.9.1475

[B33] McLaughlin J. E. Boyer J. S. (2004). Sugar-responsive gene expression, invertase activity, and senescence in aborting maize ovaries at low water potentials. *Ann. Bot.* 94 675–689. 10.1093/aob/mch193 15355866 PMC4242214

[B34] Moll R. H. Kamprath E. J. Jackson W. A. (1982). Analysis and interpretation of factors which contribute to efficiency of nitrogen-utilization. *Agr. J.* 74 562–564.

[B35] Monneveux P. Zaidi P. H. Sanchez C. (2005). Population density and low nitrogen affects yield-associated traits in tropical maize. *Crop Sci.* 45 535–545. 10.2135/cropsci2005.0535

[B36] Mueller S. M. Camberato J. J. Messina C. Shanahan J. Zhang H. Vyn T. J. (2017). Late-split nitrogen applications increased maize plant nitrogen recovery but not yield under moderate to high nitrogen rates. *Agr. J.* 109 2689–2699. 10.2134/agronj2017.05.0282

[B37] Mueller S. M. Vyn T. J. (2018a). Can late-split nitrogen application increase ear nitrogen accumulation rate during the critical period in maize? *Crop Sci.* 58 1717–1728. 10.2135/cropsci2018.02.0118

[B38] Mueller S. M. Vyn T. J. (2018b). Physiological constraints to realizing maize grain yield recovery with silking-stage nitrogen fertilizer applications. *Field Crop. Res.* 228 102–109. 10.1016/j.fcr.2018.08.025

[B39] Nasielski J. Earl H. Deen B. (2019). Luxury vegetative nitrogen uptake in maize buffers grain yield under post-silking water and nitrogen stress: a mechanistic understanding. *Front. Plant Sci.* 10:318. 10.3389/fpls.2019.00318 30972083 PMC6443847

[B40] Ning P. Yang L. Li C. J. Fritschi F. B. (2018). Post-silking carbon partitioning under nitrogen deficiency revealed sink limitation of grain yield in maize. *J. Exp. Bot.* 69 1707–1719. 10.1093/jxb/erx496 29361032 PMC5888971

[B41] Olmedo Pico L. B. Zhang C. Vyn T. J. (2019). “Nitrogen management consequences on endosperm cell number formation in maize kernels,” in *Proceedings of the ASA-CSSA-SSSA International Annual Meeting, 2019 Nov 10-13*, San Antonio, TX.

[B42] Ordonez R. A. Savin R. Cossani C. M. Slafer G. A. (2018). Maize grain weight sensitivity to source-sink manipulations under a wide range of field conditions. *Crop Sci.* 58 2542–2557. 10.2135/cropsci2017.11.0676

[B43] Oscarson P. (2000). The strategy of the wheat plant in acclimating growth and grain production to nitrogen availability. *J. Exp. Bot.* 51 1921–1929. 10.1093/jexbot/51.352.1921 11113170

[B44] Paponov I. A. Engels C. (2005). Effect of nitrogen supply on carbon and nitrogen partitioning after flowering in maize. *J. Plant Nutr. Soil Sci.* 168 447–453. 10.1002/jpln.200520505

[B45] Paponov I. A. Sambo P. Erley G. S. A. Presterl T. Geiger H. H. Engels C. (2005b). Kernel set in maize genotypes differing in nitrogen use efficiency in response to resource availability around flowering. *Plant Soil* 272 101–110. 10.1007/s11104-004-4210-4218

[B46] Paponov I. A. Sambo P. Erley G. S. A. Presterl T. Geiger H. H. Engels C. (2005a). Grain yield and kernel weight of two maize genotypes differing in nitrogen use efficiency at various levels of nitrogen and carbohydrate availability during flowering and grain filling. *Plant Soil* 272 111–123. 10.1007/s11104-004-4211-4217

[B47] Peng Y. F. Li C. J. Fritschi F. B. (2013). Apoplastic infusion of sucrose into stem internodes during female flowering does not increase grain yield in maize plants grown under nitrogen-limiting conditions. *Physiol. Plant.* 148 470–480. 10.1111/j.1399-3054.2012.01711.x 23061679

[B48] Perez C. M. Palmiano E. P. Baun L. C. Juliano B. O. (1971). Starch metabolism in leaf sheaths and culm of rice. *Plant Physiol.* 47 404–408. 10.1104/pp.47.3.404 16657631 PMC365878

[B49] Poorter H. Niklas K. J. Reich P. B. Oleksyn J. Poot P. Mommer L. (2012). Biomass allocation to leaves, stems and roots: meta-analyses of interspecific variation and environmental control. *New Phytol.* 193 30–50. 10.1111/j.1469-8137.2011.03952.x 22085245

[B50] Presterl T. Groh S. Landbeck M. Seitz G. Schmidt W. Geiger H. H. (2002). Nitrogen uptake and utilization efficiency of European maize hybrids developed under conditions of low and high nitrogen input. *Plant Breed.* 121 480–486. 10.1046/j.1439-0523.2002.00770.x

[B51] Reddy V. M. Daynard T. B. (1983). Endosperm characteristics associated with rate of grain filling and kernel size in corn. *Maydica* 28 339–355.

[B52] Sattelmacher B. Horst W. J. Becker H. C. (1994). Factors that contribute to genetic variation for nutrient efficiency of crop plants. *Z. Pflanz. Bodenkunde* 157 215–224. 10.1002/jpln.19941570309

[B53] Severini A. D. Borras L. Westgate M. E. Cirilo A. G. (2011). Kernel number and kernel weight determination in dent and popcorn maize. *Field Crop. Res.* 120 360–369. 10.1016/j.fcr.2010.11.013

[B54] Shannon J. C. (1972). Movement of 14C-labeled assimilates into kernels of *Zea mays* L: I. Pattern and rate of sugar movement. *Plant Physiol.* 49 198–202. 10.1104/pp.49.2.198 16657924 PMC365928

[B55] Takei K. Sakakibara H. Taniguchi M. Sugiyama T. (2001). Nitrogen-dependent accumulation of cytokinins in root and the translocation to leaf: implication of cytokinin species that induces gene expression of maize response regulator. *Plant Cell Physiol.* 42 85–93. 10.1093/pcp/pce009 11158447

[B56] Tollenaar M. (1977). Sink-source relationships during reproductive development in maize. A review. *Maydica* 22 49–75.

[B57] Tollenaar M. Daynard T. B. (1978). Dry weight, soluble sugar content, and starch content of maize kernels during early postsilking period. *Can. J. Plant Sci.* 58 199–206. 10.4141/cjps78-029

[B58] Triboi E. Triboi-Blondel A. M. (2002). Productivity and grain or seed composition: a new approach to an old problem - invited paper. *Eur. J. Agron.* 16 163–186. 10.1016/s1161-0301(01)00146-140

[B59] Uhart S. A. Andrade F. H. (1995). Nitrogen deficiency in maize: I. Effects on crop growth, development, dry matter partitioning, and kernel set. *Crop Sci.* 35 1376–1383. 10.2135/cropsci1995.0011183X003500050020x

[B60] Wang R. Y. Bowling L. C. Cherkauer K. A. (2016). Estimation of the effects of climate variability on crop yield in the Midwest USA. *Agr. Forest Meteorol.* 216 141–156. 10.1016/j.agrformet.2015.10.001

[B61] Yan P. Chen Y. Q. A. Sui P. Vogel A. Zhang X. P. (2018). Effect of maize plant morphology on the formation of apical kernels at different sowing dates and under different plant densities. *Field Crops Res.* 223 83–92. 10.1016/j.fcr.2018.04.008

[B62] Yu J. J. Han J. N. Wang R. F. Li X. X. (2016). Down-regulation of nitrogen/carbon metabolism coupled with coordinative hormone modulation contributes to developmental inhibition of the maize ear under nitrogen limitation. *Planta* 244 111–124. 10.1007/s00425-016-2499-2491 26979324

[B63] Zinselmeier C. Jeong B. R. Boyer J. S. (1999). Starch and the control of kernel number in maize at low water potentials. *Plant Physiol.* 121 25–35. 10.1104/pp.121.1.25 10482657 PMC59374

