# Supplementary material for: Differential Regulation of Kernel Set and Potential Kernel Weight by Nitrogen Supply and Carbohydrate Availability in Maize Genotypes Contrasting in Nitrogen Use Efficiency
Source: Front Plant Sci. 2020 May 15;11:586. doi: 10.3389/fpls.2020.00586 (PMC7243938; doi:10.3389/fpls.2020.00586)
Supplement: Supplementary file 1 [file Table_1.DOCX]

**Table S1.** Analysis of variance significance levels. Variables are plant biomass, leaf weight ratio (LWR), stalk weight ratio (SWR), root weight ratio (RWR), ear 1 and husk weight ratio (Ear+husk1WR), and ear 2 and husk weight ratio (Ear+husk2WR). The data was analyzed by 2-factor-ANOVA. The first factor was genotype (G) (GREEN, EFFI) and the second factor was treatments (T) (NN, Nn, N(N+S), nN, nn). Excluding shading from the analysis (N(N+S)), the experiment was also analyzed by 3-factor ANOVA, using factors Nv (N level before silking), Nf (N level during the lag phase) (NN, Nn, nN, nn), and G. Means and SE are presented in Figure 2. If the effect of a factor or the interaction between factors was significant, we labeled it with asterisks, * p<0.05, ** p<0.01, *** p<0.001. NS is nonsignificant at the 0.05 probability level.

| Source of variation | Biomass, g plant^-1^ | LWR, % | SWR, % | RWR, % | Ear+husk1WR, % | Ear+husk2WR, % |
| --- | --- | --- | --- | --- | --- | --- |
| Genotype (G) | NS | NS | NS | * | *** | *** |
| Treatment (T) | *** | ** | *** | * | *** | *** |
| G × T | NS | NS | NS | NS | NS | *** |
|  |  |  |  |  |  |  |
| G | NS | NS | NS | * | *** | *** |
| Nv | *** | NS | NS | NS | *** | NS |
| Nf | NS | NS | *** | NS | NS | *** |
| G × Nv | NS | NS | NS | NS | NS | * |
| G × Nf | NS | NS | NS | NS | NS | ** |
| Nv × Nf | NS | NS | ** | NS | NS | ** |
| G × Nv × Nf | NS | NS | NS | NS | NS | * |

**Table S2.** Analysis of variance significance levels. Variables are reducing sugars, sucrose, and starch in the stalk and kernels. The data was analyzed by 2-factor-ANOVA. The first factor was genotype (G) (GREEN, EFFI) and the second factor was treatments (T) (NN, Nn, N(N+S), nN, nn). Excluding shading from the analysis (N(N+S)), the experiment was also analyzed by 3-factor ANOVA, using factors Nv (N level before silking), Nf (N level during the lag phase) (NN, Nn, nN, nn), and G. Means and SE are presented in Figure 3. If the effect of a factor or the interaction between factors was significant, we labeled it with asterisks, * p<0.05, ** p<0.01, *** p<0.001. NS is nonsignificant at the 0.05 probability level.

| Source of variation | Stalk | | | Kernel | | |
| --- | --- | --- | --- | --- | --- | --- |
|  | Reducing sugars | Sucrose | Starch | Reducing sugars | Sucrose | Starch |
| Genotype (G) | * | *** | *** | NS | ** | * |
| Treatment (T) | *** | *** | *** | ** | ** | NS |
| G × T | NS | NS | NS | NS | NS | NS |
|  |  |  |  |  |  |  |
| G | NS | ** | ** | * | ** | * |
| Nv | NS | NS | *** | * | ** | NS |
| Nf | ** | NS | NS | * | * | NS |
| G × Nv | NS | NS | NS | NS | NS | NS |
| G × Nf | ** | NS | NS | NS | NS | NS |
| Nv × Nf | NS | NS | NS | NS | NS | NS |
| G × Nv × Nf | NS | NS | NS | NS | NS | NS |

**Table S3.** Analysis of variance significance levels. Variables are plant N uptake, N concentration in two genotypes with different N-use efficiency at the end of the lag phase in leaves, stalk, roots, kernels, and cob. The data was analyzed by 2-factor-ANOVA. The first factor was genotype (G) (GREEN, EFFI) and the second factor was treatments (T) (NN, Nn, N(N+S), nN, nn). Excluding shading from the analysis (N(N+S)), the experiment was also analyzed by 3-factor ANOVA, using factors Nv (N level before silking), Nf (N level during the lag phase) (NN, Nn, nN, nn), and G. Means and SE are presented in Figure 4. If the effect of a factor or the interaction between factors was significant, we labeled it with asterisks, * p<0.05, ** p<0.01, *** p<0.001. NS is nonsignificant at the 0.05 probability level.

| Source of variation | Plant N, mg plant^-1^ | Leaf N, % | Stalk N, % | Root N, % | Kernel N, % | Cob N, % |
| --- | --- | --- | --- | --- | --- | --- |
| Genotype (G) | NS | NS | NS | *** | NS | NS |
| Treatment (T) | *** | *** | *** | *** | *** | *** |
| G × T | NS | NS | * | NS | ** | NS |
|  |  |  |  |  |  |  |
| G | NS | NS | * | *** | NS | NS |
| Nv | *** | NS | NS | NS | NS | NS |
| Nf | *** | *** | * | *** | *** | *** |
| G × Nv | NS | NS | NS | NS | ** | NS |
| G × Nf | NS | NS | * | NS | NS | NS |
| Nv × Nf | *** | * | * | NS | *** | *** |
| G × Nv × Nf | NS | NS | NS | NS | NS | NS |

**Table S4.** Analysis of variance significance levels. Variables are percentage of ^15^N distribution at the end of the lag phase in two genotypes with different N-use efficiency to leaves, stalk, roots, first ear and husks, and second ear and husks. The data was analyzed by 2-factor-ANOVA. The first factor was genotype (G) (GREEN, EFFI) and the second factor was treatments (T) (NN, Nn, N(N+S), nN, nn). Excluding shading from the analysis (N(N+S)), the experiment was also analyzed by 3-factor ANOVA, using factors Nv (N level before silking), Nf (N level during the lag phase) (NN, Nn, nN, nn), and G. Means and SE are presented in Figure 5. If the effect of a factor or the interaction between factors was significant, we labeled it with asterisks, * p<0.05, ** p<0.01, *** p<0.001. NS is nonsignificant at the 0.05 probability level.

| Source of variation | Leaf ^15^N distribution, % | Stalk ^15^N distribution, % | Root ^15^N distribution, % | Ear+husk1 ^15^N distribution, % | Ear+husk2 ^15^N distribution, % |
| --- | --- | --- | --- | --- | --- |
| Genotype (G) | NS | NS | NS | *** | *** |
| Treatment (T) | ** | *** | NS | *** | *** |
| G × T | NS | NS | NS | NS | *** |
|  |  |  |  |  |  |
| G | NS | * | NS | ** | *** |
| Nv | NS | NS | NS | ** | * |
| Nf | *** | NS | NS | *** | *** |
| G × Nv | NS | NS | NS | NS | ** |
| G × Nf | NS | * | NS | NS | *** |
| Nv × Nf | NS | NS | NS | NS | *** |
| G × Nv × Nf | NS | NS | NS | NS | ** |

**Table S5.** Analysis of variance significance levels. Variables are kernel number and kernel weight for the first ear of two genotypes with different N-use efficiency grown under high or low N supply during vegetative growth or during the lag phase and under full light or shading during the lag phase. All treatments were grown under luxury conditions during effective grain filling established by decreasing plant density to 3 plants m^-2^ und un-limited N supply. Two separate 3 factor-ANOVAs were carried out. In the first analysis, the factors genotype (G), N level during vegetative stage (Nv), and N level after flowering (Nf) were tested (NN, Nn, nN, nn). In the second analysis, the factor genotype (G), N level (both during vegetative and the lag phase), and shading (S) were tested (NN, N(N+S), nn, n(n+S)). Means and SE are presented in Figure 6. If the effect of a factor or the interaction between factors was significant, we labeled it with asterisks, * p<0.05, ** p<0.01, *** p<0.001. NS is nonsignificant at the 0.05 probability level.

| Source of variation | Kernel number, plant^-1^ | Kernel weight, mg |
| --- | --- | --- |
| Genotype (G) | NS | NS |
| Nv | ** | ** |
| Nf | NS | * |
| G × Nv | ** | NS |
| G × Nf | NS | NS |
| Nv × Nf | NS | NS |
| G × Nv × Nf | NS | NS |
|  |  |  |
| G | ** | *** |
| N | *** | *** |
| S (Shading) | *** | * |
| G × N | ** | NS |
| G × S | ** | NS |
| N × S | ** | NS |
| G × N × S | NS | NS |
